# The impact of gut-liver-derived mediators on the organ crosstalk with brain, heart, and kidney: A systematic review

**DOI:** 10.1016/j.molmet.2025.102295

**Published:** 2025-11-29

**Authors:** Shruti Bhargava, Zhuangting Rao, Raymond Vanholder, Frank Tacke, Heidi Noels, Vera Jankowski, Juliane Hermann, Joachim Jankowski

**Affiliations:** 1 Institute of Molecular Cardiovascular Research, Medical Faculty, RWTH Aachen University, Germany; 2 Nephrology Section, Department of Internal Medicine and Pediatrics, University Hospital, Ghent, Belgium; 3 Department of Hepatology and Gastroenterology, Charité - Universitätsmedizin Berlin, Campus Virchow-Klinikum (CVK) and Campus Charité Mitte (CCM), Berlin, Germany; 4 Aachen-Maastricht Institute for Cardiorenal Disease (AMICARE), University Hospital RWTH Aachen, Aachen, Germany; 5 Experimental Vascular Pathology, Cardiovascular Research Institute Maastricht (CARIM), University of Maastricht, the Netherlands

**Keywords:** Gut-liver axis, Mediators of organ crosstalk, Gut-liver-kidney axis, Gut-liver-brain axis, Gut-liver-heart axis, Mechanisms of organ crosstalk

## Abstract

**Introduction:**

The current understanding of interactions and crosstalk among essential organs remains incomplete, mainly due to the limitations of studies on the systemic mechanisms at play. The gut and the liver are essential for the functioning of the entire body, and their derived mediators circulate through blood or lymph, impacting other organs like the brain, heart, and kidneys.

**Aim:**

This publication reviews gut-liver-derived mediators, which were tested and validated *in vivo* in humans and rodents, together with the current knowledge of their systemic effects on key vital organs.

**Method:**

Original articles published up to February 2025, based on clinical trials or in vivo experimental models, were retrieved from PubMed and Web of Science.

**Results:**

During this systematic analysis, 28 gut-liver-derived mediators were identified from 52 publications and classified into five distinct groups based on their molecular characteristics: (a) low molecular weight metabolites, (b) endotoxins, (c) hormones, (d) lipids and (e) proteins. Additionally, the mechanism of action for each of these molecules was specified, aimed at providing a mechanistic overview of their effects on the brain, heart, and kidneys.

**Discussion:**

The diverse and occasionally conflicting impact of the identified mediators on comorbidities necessitates further investigations pinpointing key mechanisms influencing disease genesis and progression.

**Conclusion:**

Our research shows the necessity of a thorough examination of these mediators, exploring their diagnostic and therapeutic potential in a holistic multi-organ setting, to elucidate inter-organ crosstalk.

## Highlights

• Review highlights gut-liver pathway gaps and urges prioritizing inter-organ science to enable more holistic disease therapy.
• 28 gut–liver mediators were identified and grouped by molecule type, with their effects on brain, heart, and kidneys reviewed.
• Review exposes gaps in gut-liver pathways affecting organs and urges advancing inter-organ science to enable holistic therapies.

## 1 Introduction

The reciprocal interaction between the gut and the liver, referred to as the ‘gut-liver axis,’ involves the exchange of signals mediated by various factors, which, in turn, are influenced by elements such as diet and environmental elements [1]. The gut-liver axis consists of anatomical connections (e.g., splanchnic veins draining blood from the intestine to the liver, bile ducts draining bile from the liver to the intestine), and soluble mediators and cellular components in both organs sensing and responding to these mediators [2].

Under physiologic conditions, the intestinal mucosa and vascular barrier prevent dysregulation of intestinal microbiota and protect the circulation against harmful intestinal content [3]. Mediators like microbial antigens [4], metabolites [5] and bile acids [6] maintain the gut and liver structure and function. However, under pathologic conditions, proinflammatory factors such as tumour necrosis factor-alpha (TNF-α) easily cross the intestinal mucosal and vascular barrier and intensify the progression of liver inflammation [7,8]. The intricate and multifaceted interactions among diverse mediators within the gastrointestinal tract and the liver collectively determine the homeostatic equilibrium between these two vital organs [9]. Moreover, the gut-liver axis also impacts other vital organs, such as the brain [10], the heart [11], and the kidneys [12].

The clinical relevance of organ crosstalk is well established, as demonstrated by the strong connection between metabolic dysfunction-associated steatotic liver disease and cardiovascular-renal disease [13]. On a mechanistic level, the interactions between these vital organs are not completely understood due to a lack of systematic studies focusing on the impact of mediators from the gut-liver axis on other vital organs [3,9]. Understanding organ–crosstalk interactions is important because they account for complex interactions [14] and systemic effects [15], with the potential that interfering with these, may lead to more effective and precise therapeutic interventions. Due to patho-physiologic and metabolic complexity, immune interactions, and tissue-specific factors, *in vivo* studies are the only possibility to depict the real-time impact of mediators in the body. As an example, deoxycholic acid exhibits pro-inflammatory and cytotoxic effects on colonic epithelial cells *in vitro* [16]. However, under physiological conditions, these effects are moderated by enterohepatic circulation and sulfation via hepatic sulfotransferases, which enhance its solubility and promote excretion [17]. This metabolic regulation would limit its cytotoxicity *in vivo,* which cannot be directly quantified. Therefore, this systematic review focused on mediators validated *in vivo* or in humans. In this study, targeting shared signalling nodes, where gut-liver-derived mediators converge, is hypothesised to yield greater therapeutic benefit across vital organs than organ-isolated approaches. A conceptual framework that organises mediator classes and maps their cross-organ effects onto a set of convergent signalling pathways, prioritises pathway-level drug targets and system-wide biomarkers. Focusing on these intersections enables multi-target, cross-organ trial designs and endpoints.

## 2 Materials and methods

### 2.1 Literature search

This systematic review followed standard PRISMA guidelines and was registered on PROSPERO with the registration ID ‘CRD42023403419’ [18]. The literature search was conducted on PubMed [19] and Web of Science [20] databases, and articles published before February 2025 were included in this review. The initial literature search was carried out using the search terms “gut”, “liver, “gut-liver axis”, “mediator”, “*in vivo*”, and “clinical trial” in different systematic combinations, as summarised in Table 1. The titles were evaluated, and abstracts of all the retrieved original articles were further reviewed based on predefined inclusion and exclusion criteria (see below).

**Table 1:** Databases used for collecting articles.

Types of databases	Keywords	Search strategy	Filters used	Number of records identified
**PubMed**	Gut, liver, gut-liver axis, mediator, *in vivo*, clinical trial	((gut) AND (liver)) AND (mediator) AND (clinical trial)	Published before February 2025	66
		((gut) AND (liver)) AND (mediator) AND (*in vivo*)		401
		(gut liver axis) AND (clinical trial)		62
		(gut liver axis) AND (mediators) AND (clinical trial)		6
		((gut liver axis) AND (mediators)) AND (*in vivo*)		58
				Total:593
**Web of science**	Gut, liver, gut-liver axis, mediator, *in vivo*, clinical trial	((gut) AND (liver)) AND (mediator) AND (clinical trial)		37
		((gut) AND (liver)) AND (mediator) AND (*in vivo*)		76
		(gut liver axis) AND (clinical trial)		210
		(gut liver axis) AND (mediator) AND (clinical trial)		10
		((gut liver axis) AND (mediator)) AND (*in vivo*)		10
				Total:343

### 2.2 Inclusion and exclusion criteria

Articles that met the following criteria were included: (a) published in the English language, (b) original articles and (c) clinical trials or *in vivo* experimental studies (rodents). Review papers, abstracts or conference proceedings, articles containing repetitive data, case reports, and studies that solely relied on non-rodent animal models were excluded.

### 2.3 Data extraction, analysis and synthesis

Two authors (SB, ZTR) individually examined the literature and evaluated articles according to the predefined inclusion criteria to alleviate selection bias. This evaluation encompassed all titles and abstracts to determine their eligibility for analysis. A third reviewer (JJ) resolved possible discordances. Studies that did not meet the predetermined criteria were excluded from further consideration. The ultimately retrieved articles were read, and information regarding the function of mediators of the gut-liver axis was extracted. The identified mediators, extracted as data, were classified based on their molecular characteristics. The extracted data were imported into a table format for easy access and visualisation (Table 2).

**Table 2:** Mediators released from the gut-liver axis mentioned in search results.

Classifi-cation	Mediator	Gut-liver axis	Reference
Low molecular weight metabolites	Bile compounds	• Shapes intestinal bacterial profiles	Bajaj et al., 2019 [35]
		• Alleviates hypercholesterolemia	Gaillard et al., 2021 [36]
		• Causes inflammation in the intestine	Zhao et al., 2021 [39]
		• Causes hepatic disease	Owen et al., 2010 [40] Spivak et al., 2025 [37]
		• Accelerates fat metabolism	Druart et al., 2013 [41]
		• Facilitates digestion	Koelfat et al., 2021 [38]
	Butyrate	• Reduces NAFLD	Endo et al., 2013 [22]
		• Reduces atherosclerosis • Improves intestinal barrier • Induces apoptosis of proinflammatory macrophages • Rectifies immune dysregulation	Du et al., 2020 [26] Zhao et al., 2024 [24] Sarkar et al., 2023 [23] Wang et al., 2024 [25]
	Histamine	• Reduces plasma IGF-1	Liao et al., 1999 [27]
	Indole-3-acetic acid	• Alleviates NAFLD	Ji et al., 2019 [28]
	Indole	• Alleviates liver inflammation	Beaumont et al., 2018 [29]
	Lactate	• Induction of the fibrotic pathway	Sarkar et al., 2020 [30]
		• D-lactate promotes liver pathogen clearance • D- lactate controls vascular traffic • Lactic acid alleviates lipid metabolism disorders	Mcdonald et al., 2020 [32] Zucoloto et al., 2023 [176] Zhao et al., 2025 [31]
	p-Cresol Phenylpropionic acid	• Improves glucose homeostasis • Reduces CYP2E1-mediated liver injury	Brial et al., 2020 [33] Cho et al., 2023 [34]
	Vitamin A Vitamin B	• Promotes inflammatory migration of gut • Maintains amino acid metabolism	Neumann et al., 2012 [42] Shen et al., 2025 [43]
	Vitamin D	• Improves inflammation of the intestinal/liver and hepatic steatosis	Su et al., 2018 [44]
		• Promotes calcium absorption	Reynokls et al., 2020 [45]
Endotoxin	Lipopolysaccharide	• Oxidative damage to liver cells • Induces gut inflammation	McCuskey et al., 1995 [167] Von Baehr et al., 2000 [177]
		• Induces hepatitis	Akiba et al., 2020 [178] Lin et al., 2012 [179]
	Lipoteichoic acid	• Induces pancreatitis • Increased in advanced chronic liver disease	Vonlaufen et al., 2007 [168] Simbrunner et al., 2023 [180]
Hormones	Glucagon-like peptide −1	• Suppresses hepatic lipogenesis	Ben-Shlomo et al., 2011 [92]
	Neuropeptide Y	• Reduces portal hypertension	Moleda et al., 2011 [96]
	Norepinephrine	• Induces hepatocellular dysfunction	Yang et al., 2001 [95]
	Metabolitin	• Inhibits intestinal fat absorption and improves NAFLD	Teng et al., 2020 [94]
	Serotonin	• Assists liver regeneration	Svejda et al., 2013 [93]
Lipids	2-Oleoylglycerol Ceramide	• Promote liver inflammation and hepatic fibrosis • Induces obesity and insulin resistance	Yang et al., 2023 [123] Xie et al., 2017 [115]
	Sphingosine-1-phosphate	• Induces gut inflammation	Chu et al., 2021 [116]
Protein	Cathepsin K	• Stimulates intestinal tumour metastasis	Li et al., 2019 [125]
	Fibroblast growth factor	• FGF 15/19 improves fatty liver regeneration	Alvarez-Sola et al., 2017 [126]
		• FGF 15/19 regulates bile acids to facilitate digestion	Koelfat et al., 2021 [38]
		• FGF 15/19 reduces liver fibrosis	Schumacher et al., 2020 [127] Simbrunner et al. [129]
		• FGF 21 alleviates hepatic and intestinal damage in NAFLD	Lin et al., 2023 [128]
	Lipopolysaccharide-binding protein	• Promotes the inflammatory response of LPS	Molinaro et al., 2020 [130]
	Myeloid differentiation primary response 88	• Excites inflammation-associated cytokines • Inflammatory response	Molinaro et al., 2020 [130] Wang et al., 2024 [131]
	Receptor-interacting protein kinase 3	• Mediates autoimmune hepatitis	Zhang et al., 2021 [132]
	TIM3	• Enhances anti-bacterial immunity	Riva et al., 2021 [133]
	TNF-α	• Induces gut inflammation	Von Baehr et al., 2000 [177]
		• Induces insulin resistance	Lang et al., 1992 [134]

Next, using the names of the retrieved mediators as search keywords, an exploration of associations with the brain, heart, and kidneys was conducted on PubMed. The same criteria were applied for the initial search. Each mediator was then considered for its impact on the brain (Table 3), the heart (Table 4) and the kidneys (Table 5). Because of space limitations and the major patho-physiologic role of these three organs, this publication will essentially concentrate on the effects beyond the gut-liver axis.

**Table 3:** Mediators signalling from the gut-liver axis to the brain.

Classifi-cation	Mediator	Gut-liver axis
	Bile acids [35,36]	• Regulate appetite and satiety [46]
Low molecular weight metabolites	Butyrate [[22], [23], [24], [25], [26]]	• Improves energy metabolism [54]
	Histamine [27]	• Enhances vascular permeability [58]
	Indole-3-acetic acid [28]	• Inhibits neuroinflammation [62]
	Indole [29]	• Inhibits neuroinflammation [62]
	Lactate [30]	• Long-term memory formation [68]
	p-Cresol [33] Phenylpropionic acid [34]	• Alters neurotransmitter signalling [72] • Inhibits gastric acid secretion [76]
	Vitamin A [42] Vitamin B [43]	• Reduces apoptosis [77] • Preserves cortical structure [79]
	Vitamin D [44,45]	• Reduces cerebral ischemia [83]
Endotoxin	Lipopolysaccharide [168,178,179] Lipoteichoic acid [180]	• Causes neuro-dysfunction [86] • Causes immune activation [90] • Disrupts blood–brain-barrier [90]
Hormones	Glucagon-like peptide-1 [92]	• Improves memory formation [98]
	Neuropeptide Y [96]	• Enhances the post-traumatic recovery [108]
	Norepinephrine [95]	• Enhances visual attention [104]
	Serotonin [93]	• Regulates motor activity [112]
Lipids	2-Oleoylglycerol [123] Ceramide [115]	• Synaptic plasticity agonist [124] • Increases depression [117]
	Sphingosine-1-phosphate [116]	• Cognitive impairment [120]
Protein	Cathepsin K [125]	• Central nervous system development [135]
	Fibroblast growth factor 15/19 [38,126,127]	• Promotes neurogenesis [139]
	Fibroblast growth factor 21 [128]	• Reduces neuroinflammation and oxidant stress [140]
	Lipopolysaccharide-binding protein [130]	• Induces depression symptoms [143]
	Myeloid differentiation primary response 88 [130]	• Mediates apoptosis and inflammation [146]
	Receptor-interacting protein kinase 3 [132]	• Programmed necrosis [150]
	TIM3 [133]	• Upregulated in severe traumatic brain injury [153]
	TNF-α [134,177]	• Development of astrocytes [159]

**Table 4:** Mediators signalling from the gut-liver axis to the heart and vascular system.

Classifi-cation	Mediator	In the heart and vascular system
Low molecular weight metabolites	Bile compounds [35,36,38]	• Protects against heart failure [47]
		• Mediates atherosclerosis [52]
	Butyrate [[22], [23], [24], [25], [26]]	• Prevents hypertrophy [55]
	Histamine [27]	• Enhances vascular permeability [59]
	Indole-3-acetic acid [28]	• Induces cardiac hypertrophy [64]
	Indole [29]	• Lowers heart rate [63]
	Lactate [30] p-cresol [33] Phenylpropionic acid [34]	• Accelerates vascular permeability [69] • Vascular remodelling [73,74] • Enhances CVD risk [75]
	Vitamin A [42] Vitamin B [43]	• Promotes aortic valve stenosis [78] • Reduces CVD risk [80]
	Vitamin D [44,45]	• Reduces arterial stiffness and cardiac afterload [84]
Endotoxin	Lipopolysaccharide [168,178,179] Lipoteichoic acid [180]	• Aggravates cardiomyocyte injury [87] • Causes cardiac depression [91] • Induces coronary vascular disturbances [91]
Hormones	Glucagon-like peptide-1[92]	• Enhances myocardial functions [100]
	Neuropeptide Y [96]	• Reduces inflammation and fibrosis [109]
	Norepinephrine [95]	• Increases ischemic vulnerability [105]
	Serotonin [93]	• Induces heart valve disease [113]
Lipids	Ceramide [115]	• Mediates vascular dysfunction [118]
	Sphingosine-1-phosphate [116]	• Contributes to cardiac dysfunction [121]
Protein	Cathepsin K [125]	• Endothelial inflammation and vascular remodelling [136]
	Fibroblast growth factor 15/19 [38,126,127]	• Protects the heart from oxidative stress [141]
	Lipopolysaccharide-binding protein [130]	• Improves cardiomyocyte contractility [144]
	Myeloid differentiation primary response 88 [130]	• Causes cardiac hypertrophy [148]
	Receptor-interacting protein kinase 3 [132]	• Causes cardiomyocyte necroptosis [151]
	TIM3	• Contributes to coronary heart disease [156]
	TNF-α [134,177]	• Heart failure [162]

**Table 5:** Mediators signalling from gut-liver axis to kidney.

Classifi-cation	Mediator	Gut-liver-kidney axis
Low molecular weight metabolites	Bile acids [35,36]	• Mediates water homeostasis [51]
		• Prevents CKD [50]
	Butyrate [[22], [23], [24], [25], [26]]	• Protects against kidney disease [56]
	Histamine [27]	• Contributes to renal ischemia [60]
	Indole-3-acetic acid [28]	• Induces renal inflammation [65]
	Indole [29]	• Accelerates CKD progression [66]
	Lactate [30]	• Aggravates septic acute renal injury [70]
	p-Cresol [33]	• Causes renal fibrosis [73]
	Vitamin B [43]	• Improves peripheral polyneuropathy [81]
	Vitamin D [44,45]	• Attenuates kidney disease [85]
Endotoxin	Lipopolysaccharide [168,178,179] Lipoteichoic acid [180]	• Induces kidney dysfunction [88] • Protects against renal injury [89]
Hormones	Glucagon-like peptide-1 [92]	• Regulates sodium balance [102]
	Neuropeptide Y [96]	• Alleviates renal necroinflammation [110]
	Norepinephrine [95]	• Renal Na + absorption [106]
	Serotonin [93]	• Promotes vasodilation [114]
Lipids	Ceramide [115]	• Aggravates renal interstitial fibrosis [119]
	Sphingosine-1-phosphate [116]	• Enhance inflammation and fibrosis [122]
Protein	Cathepsin K [125]	• Kidney remodelling and hypertension [137]
	Fibroblast growth factor 21 [128]	• Reduces oxidative stress [142]
	Lipopolysaccharide-binding protein [130]	• Activates TLR-4 [145]
	Myeloid differentiation primary response 88 [130]	• Promotes renal inflammation [147]
	Receptor-interacting protein kinase 3 [132]	• Enhances mitochondrial dysfunction [152]
	TNF-α [134,177]	• Promotes CKD [161]

## 3 Results

**Figure 1:**
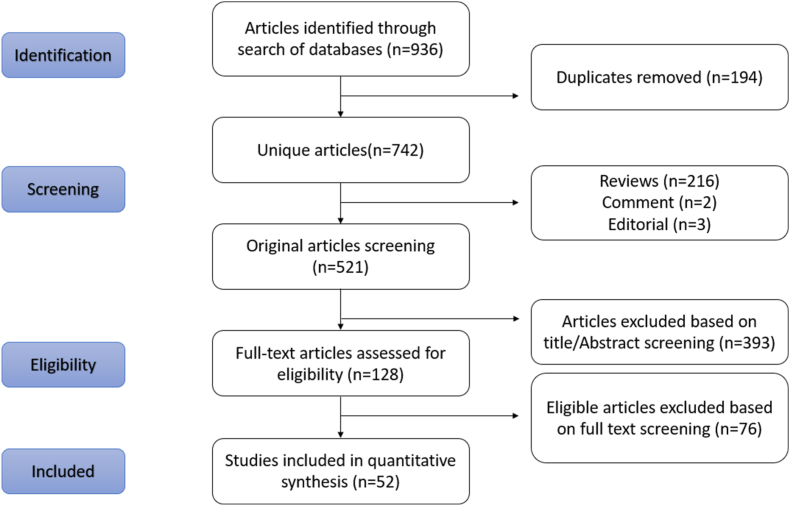
PRISMA flow diagram.

The initial literature search resulted in 936 studies (Figure 1). After initial screening, 194 duplicates, 216 reviews, two comments and three editorials were excluded from further analysis. The titles and abstracts of the remaining 521 papers were screened, and 128 full-text articles were shortlisted for further review. After full-text screening, irrelevant articles (*n* = 76) were excluded, resulting in 52 articles for in-depth analysis.

**Figure 2:**
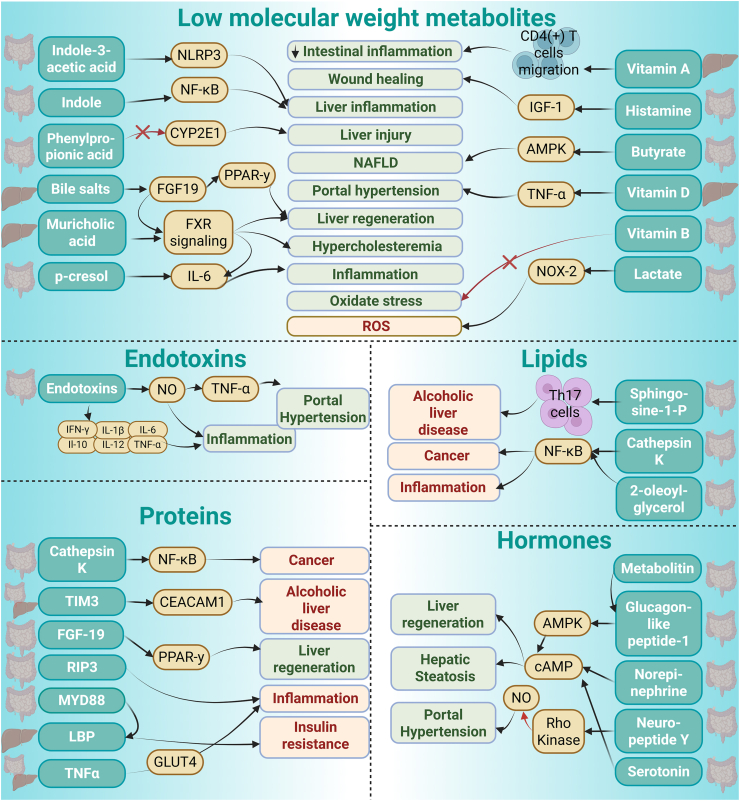
Gut liver mediators as different classes (i) Low molecular weight metabolites (ii) Endotoxins (iii) Lipids (iv) Hormones (v) proteins and their impact on the gut-liver axis.

In the course of the systematic analysis, the mediators were listed as shown in Table 2 and classified into five distinct groups: (a) low molecular weight metabolites (MW < 1,000Da [21]), (b) endotoxins, (c) hormones, (d) lipids and (e) proteins. The mechanisms by which they impact the gut-liver axis are elaborated in **Supplementary data 1.** The impact of the gut-liver mediators identified in this systematic review is visualised in Figure 2.

### 3.1 Low molecular weight intestinal metabolites (MW < 1.000Da)

**Figure 3:**
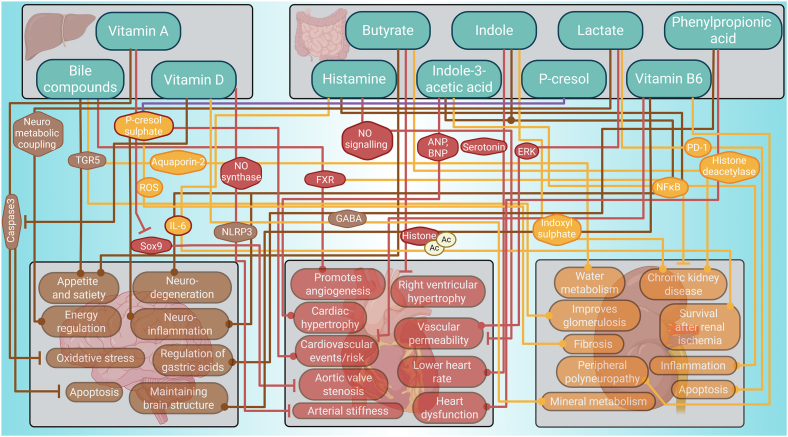
Low molecular weight metabolites exert an impact over the brain, heart and kidneys.

‘Low molecular weight metabolites’ play crucial roles in modulating liver function and metabolic processes, many of which contribute to maintaining gut-liver health [[22], [23], [24], [25], [26], [27], [28], [29], [30], [31], [32], [33], [34]], primarily playing a crucial role in preventing inflammation along this axis. This category includes metabolites with a molecular weight below 1,000Da, such as (i) bile compounds consisting of bile acids [[35], [36], [37]], their conjugated products, such as bile salts [38], and their specific constituents, such as glycocholic-acid [39], lithocholic acid [40], and their conjugated isomers [41], (ii) butyrate [[22], [23], [24], [25], [26]], (iii) histamine [27], (iv) indole-3-acetic-acid [28], (v) indole [29], (vi) lactate [[30], [31], [32]], (vii) p-cresol [33], (viii) phenylpropionic acid (PPA) [34], (ix) vitamin A [42], (x) vitamin B 6 [43] and (xi) different structural variants of vitamin D [44,45] and their impact on the heart, the brain and the kidneys is depicted in Figure 3.

Bile compounds, synthesised by the liver, regulate appetite and satiety via negative feedback through the G protein-coupled bile acid receptor (TGR5), situated on the surface of orexigenic agouti-related peptide (AgRP)/NPY neurons in the hypothalamus [46]. Bile acid- Farnesoid X Receptor (FXR) axis activation by FXR overexpression in the heart improves adipose tissue-derived mesenchymal stem cells (ADSC) survival by upregulating Nqo-1, enhancing paracrine angiogenesis by increasing Angptl4 expression and secretion, promoting angiogenesis, ameliorating cardiomyocyte apoptosis, and improving post-myocardial infarction heart failure [47]. Bile acids may also impact cardiomyocytes via TGR5 and the vitamin-D receptor, explaining their cardiotoxic (mainly hydrophobic bile acids) and cardioprotective (mainly hydrophilic bile acids) effects in heart failure [48,49]. In the kidneys, bile acids modulate pathophysiology through FXR and TGR5 activation [50,51]. FXR reduces TNFα and downstream Nuclear factor kappa-light-chain-enhancer of activated B cells (NF-κB), not only successfully halting acute kidney injury progression to chronic kidney disease (CKD) but also improving glomerulosclerosis and interstitial fibrosis while suppressing the expression of fibrogenic genes [50]. Lithocholic acid, a monohydroxy bile acid, activates TGR5, enhancing kidney aquaporin-2 expression and ameliorating impaired urinary concentration, highlighting TGR5’s involvement in kidney water handling [51]. FXR also plays a role in atherogenesis [52]. Therefore, bile compounds exert their effects indirectly through the activation of FXR and TGR5.

Butyrate, mainly produced in the gut [53], exerts protective effects on the brain, heart, and kidneys [[54], [55], [56]], e.g. by directly modulating the gut–brain axis to promote satiety, enhance energy metabolism, reduce energy intake, increase fat oxidation, and activate brown adipose tissue to oxidise fat [54]. It also prevents hypoxia-induced right ventricular hypertrophy, increases histone acetylation [55], and hinders kidney disease progression by protecting podocytes through mechanisms dependent on histone deacetylases and the activation of G protein receptor 109a [56]. Therefore, butyrate directly regulates metabolic processes via the brain while preventing cardiac hypertrophy and hindering CKD progression indirectly through histone acetylation and deacetylation.

Histamine, produced in the gut with receptors in the liver [57], activates brain microglia, releasing TNF-α and interleukin-6 (IL-6) through the signalling pathways of H1 receptor and H4 receptor-mitogen-activated protein kinase (MAPK) and phosphatidylinositol 3-kinase (PI3K) and through protein kinase B (AKT1)–NF–κB, contributing to chronic neurodegenerative disease symptoms [58]. In the heart, it promotes NO-dependent vascular dilation and permeability, disrupting the endothelial barrier via the protein kinase C/Rho-associated protein kinase/NO pathway [59]. Histamine significantly decreases animal survival following kidney ischemia through IL-6 and VEGF mRNA expression [60]. Although the mechanisms through which histamine affects the brain, heart, and kidneys differ, it indirectly regulates crucial functions in all three organs.

Indole and indole-3-acetic acid, often produced by gut microbiota [61], influence the brain by indirectly mitigating neuroinflammation through aryl hydrocarbon receptor (AhR) upregulation, NF-κB inhibition, and prevention of nucleotide-binding oligomerisation domain-like receptor family pyrin domain containing 3 (NLRP3) inflammasome formation, thereby reducing the release of inflammatory cytokines, including TNF-α and IL-6 [62]. Indole administration into the brain’s lateral ventricle leads to a lower heart rate through serotonin signalling [63]. However, indole-3-acetic acid adversely affects the heart by inducing cardiac hypertrophy and reducing diastolic function, as illustrated by the upregulation of atrial natriuretic peptide, brain natriuretic peptide, and β-myosin heavy chain [64]. In the kidneys, NF-κB is activated, inducing inflammation. Specifically, activating the aryl hydrocarbon receptor/p38 MAPK/NF-κB pathway prompts the nuclear translocation of the aryl hydrocarbon receptor complex-ligand. This, in turn, upregulates the proinflammatory enzyme cyclooxygenase-2 and stimulates the production of endothelial reactive oxygen species [65]. In kidney failure, elevated faecal indole and its toxic derivative indoxyl sulphate accumulate, worsening CKD progression [66]. Therefore, the two indoles retrieved by the current literature search have indirect positive effects on the brain; however, in the heart, the indirect effects range from regulatory to detrimental and in the kidney, they have a strong indirect negative impact [[62], [63], [64], [65], [66], [67]].

Lactate, which is produced by intestinal microbiomes-mediates neurometabolic coupling and provides the necessary energy for local protein synthesis, degradation, and signalling, regulating long-term memory function [68]. Furthermore, lactate triggers vascular permeability by disruption of VE-cadherin integrity of the endothelial cell membrane via ERK phosphorylation [69]. In septic acute kidney injury, lactate-induced activation of the PD-1/PD-L1 pathway induces immunosuppression by promoting lymphocyte apoptosis [70]. Thus, the roles of lactate vary across the three organs, indirectly regulating long-term memory in the brain, vascular permeability in the heart and controlling apoptosis in the kidneys.

P-cresol is generated by the intestinal microbiota from the amino acids tyrosine and phenylalanine and is, after intestinal absorption, metabolised by the liver to p-cresol sulfate, which has negative cardiovascular and kidney effects. In the cardiovascular system, p-cresol sulfate levels are directly associated with cardiovascular events; however, the mechanisms underlying this association are only partly understood [71]. P-cresol sulfate can cross the blood–brain barrier and promote neuroinflammation and oxidative stress, leading to alterations in neurotransmitter signalling and neuronal function, potentially contributing to cognitive and behavioural changes [72]. P-cresol sulfate from the liver accumulates in kidney cells, increasing NADPH oxidase activity and ROS production, which triggers the induction of inflammatory cytokines, participates in kidney fibrosis, and ultimately causes nephrotoxicity [73,74]. Therefore, p-cresol is indirectly toxic to the heart, the brain and the kidneys.

PPA produced by gut microbes reduces the expression of cytochrome P450 2E1 (CYP2E1) in the liver [34]. While the effect of PPA on the heart is not completely understood, its downstream metabolite phenylacetylglutamine acts via host G protein-coupled receptors, including a2A, a2B, and β2-adrenergic receptors, and is indirectly associated with a higher CVD risk [75]. We could not retrieve any references on the effect of PPA on the kidney. In the brain, γ-Aminobutyric acid (GABA) mechanisms are activated through 3-amino-3-phenylpropionic acid, which indirectly inhibits central regulation of gastric acid secretion [76]. Overall, the exact mechanism of action of PPA remains, to a large extent, unexplored.

By converting vitamin A into retinoic acid, liver sinusoidal endothelial cells prime CD4+ T-cells for migrating from the liver to the gut, potentially reducing intestinal inflammation [42]. Vitamin A impacts brain apoptosis and heart valve stenosis [77,78] while its impact on the kidney is more complex. Its metabolite, retinoic acid, is neuroprotective by releasing apoptosis-related proteins, such as caspase-3, cleaved-caspase-3 and cytochrome-c and reducing oxidative stress. In addition, it preserves the interaction between thioredoxin and apoptosis signal-regulating kinase 1 (ASK1) [77]. Overdoses of vitamin A result in retinoic acid receptor/retinoid X receptor-dependent suppression of Sox9, leading to aortic valve stenosis and leaflet calcification [78]. Therefore, while vitamin A exerts beneficial effects on the brain, elevated concentrations may exert cardiotoxic effects.

Vitamin B6, which is partly produced by gut microbiota, has a positive impact on the brain by supporting the preservation of brain surface structure [79]. Higher dietary vitamin B6 can reduce cardiovascular disease risk in men (due to beneficial changes of plasma homocysteine and estrogen levels) [80]. In patients with kidney insufficiency, it is hypothesised that vitamin B6 improves peripheral polyneuropathy by replenishing the pyridoxal-5′-phosphate levels [81]. Therefore, vitamin B6 directly supports brain structure, indirectly reduces cardiovascular risk in men, and indirectly alleviates peripheral polyneuropathy in kidney insufficiency.

Liver produces 25-hydroxyvitamin-D [82] and the metabolite D3-3β-glucuronic acid of vitamin-D-25-Hydroxyvitamin stimulates the vitamin-D-receptor-mediated pathway in the colon, primarily through apical entry after cleavage by gut bacteria [45]. Vitamin D reduces the apoptosis rate in hippocampal neurons by downregulating the expression of caspase-3 and Bax genes [83]. In the heart, vitamin D reduces arterial stiffness and cardiac afterload by stimulating nitric oxide synthase transcription, leading to higher NO production and initiating smooth muscle cell relaxation [84]. The deficiency of the active form of vitamin D, 1,25 diOH Vit D, is common among patients with CKD and contributes to disturbances in mineral metabolism, as well as an increased risk of cardiovascular complications. Consequently, supplementation with vitamin D analogues is a widely used therapeutic strategy in CKD management, aiming to restore mineral homeostasis and attenuate inflammation-associated damage [85]. Overall, vitamin D indirectly supports neuronal survival, indirectly improves cardiovascular health, and 1,25 diOH Vit D is commonly supplemented in CKD to directly counteract deficiency-related complications and inflammation.

In summary, low molecular weight metabolites significantly influence the gut-liver-brain-heart-kidney axis by modulating inflammation, metabolism, apoptosis, and vascular function through diverse, often overlapping mechanisms. While many of these metabolites exert protective effects, others-depending on their concentration, downstream products, or metabolic variants, can contribute to tissue damage and disease progression, highlighting their dualistic roles in systemic health and disease.

### 3.2 Endotoxins

**Figure 4:**
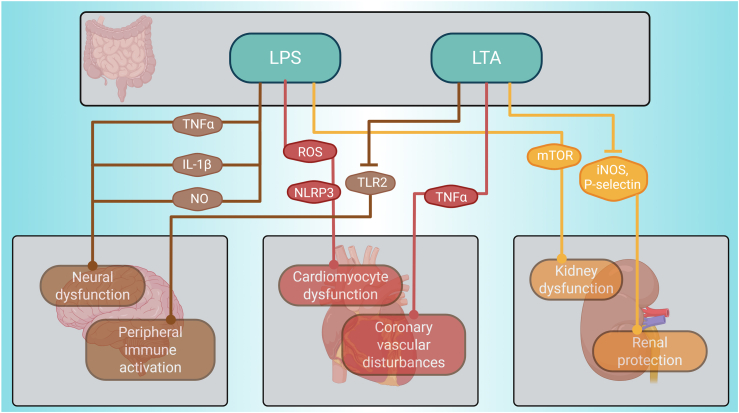
Endotoxins exert an impact over the brain, heart and kidneys.

The second category of gut-liver mediators belongs to the group of endotoxins, predominantly lipopolysaccharides (LPS) and lipoteichoic acid (LTA), which are primarily produced by the gut microbes and their impact on the heart, the brain and the kidneys is depicted in Figure 4. Endotoxins have a profound and multifaceted impact on the gut-liver axis, promoting hepatic inflammation and oxidative stress. These effects contribute not only to liver injury and fibrosis but also to the modulation of systemic inflammatory responses.

Endotoxins indirectly affect the brain and the heart negatively. LPS induces neural dysfunction by activating pathways similar to those in the gut-liver axis by inducing TNF-α, interleukin-1β (IL-1β), prostaglandin E2 (PGE2), and nitric oxide (NO) [86]. Additionally, LPS worsens cardiomyocyte dysfunction via ROS-dependent NLRP3 inflammasome-mediated pyroptosis [87]. LPS indirectly triggers kidney dysfunction by instigating a chronic inflammatory response and subsequent kidney fibrosis, partly by activated macrophages through the mammalian target of the rapamycin (mTOR) signalling pathway [88]. While LPS, which originates from gram-negative bacteria, negatively impacts the kidney, LTA, generated by Staphylococcus aureus, has a positive indirect impact on the kidneys. LTA reduces P-selectin and iNOS expression and polymorphonuclear cell recruitment, leading to lower NO, ROS and peroxynitrite production [89]. However, LTA also indirectly triggers peripheral immune activation by acting as an agonist for TLR2, initiating neuroinflammatory processes and disrupting the blood–brain barrier [90]. In the heart, LTA indirectly promotes myocardial TNF-α synthesis via CD14 [91] and activates Cox-2-dependent thromboxane A2 synthesis, leading to coronary vascular disturbances [91]. Therefore, although the majority of endotoxin-regulated processes are detrimental, LTA specifically derived from Staphylococcus aureus has been shown to exert protective effects on the kidneys.

### 3.3 Hormones

**Figure 5:**
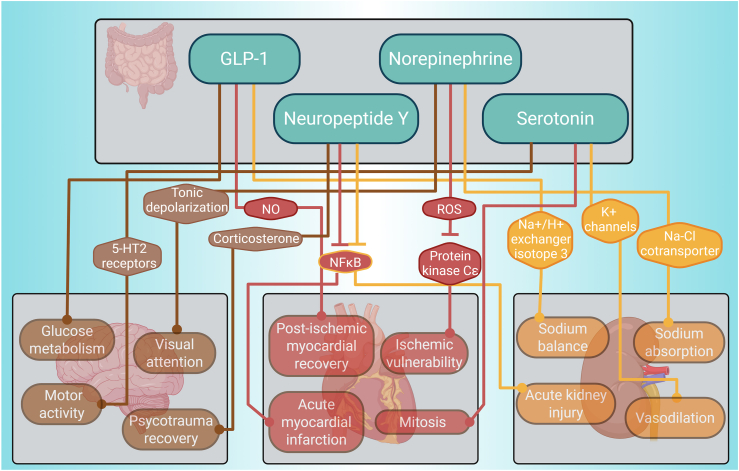
Lipids exert an impact over the brain, heart and kidneys.

The third category of retrieved gut-liver mediators includes several hormones which play a major role in the gut-liver axis by regulating liver metabolism and gut and liver inflammation by common mechanisms such as cAMP regulation and stimulation of the expression of other hormones [[92], [93], [94]]. The hormones retrieved through the systematic search include: (i) glucagon-like peptide-1 (GLP-1) [92], (ii) serotonin [93], (iii) norepinephrin [95], (iv) neuropeptide Y (NPY) [96] and their impact on the heart, the brain and the kidneys is depicted in Figure 5.

GLP-1, produced by specialised intestinal neuroendocrine cells in response to dietary components [97], regulates brain glucose metabolism and neuronal function by maintaining energy homeostasis in astrocytes [98]. The absence of GLP-1R signalling in astrocytes directly triggers an adaptive stress response that enhances systemic glucose regulation, improves memory function, preserves mitochondrial integrity, and increases Fibroblast Growth Factor-21 production [98]. In addition, GLP-1 receptor agonists directly reduce appetite and addictive drug-seeking. However, it is incompletely understood how these agents reach discrete brain sites relevant to the regulation of energy homeostasis [99]. In the heart, GLP-1 indirectly enhances post-ischemic myocardial recovery by increasing nitric oxide production and GLUT-1 translocation and improves left ventricular contractility and mitochondrial respiratory capacity after infarction [100], with beneficial cardiovascular effects even in non-diabetic patients [101]. GLP-1 and GLP-1R indirectly regulate sodium balance in the kidneys through ‘Na^+^/H^+^ exchanger isotope 3’ activity in the proximal tubule [102] and are nephroprotective in murine diabetic nephropathy [103]. GLP-1 regulates brain glucose metabolism, enhancing cardiac function and recovery and maintains kidney sodium balance.

‘Norepinephrine’ is in part produced by the gut microbes [57], and is necessary for tonic depolarization during locomotion, indirectly improving visual attention by increasing the signal-to-noise ratio of excitatory neurons, contributing to cognitive processes such as attention [104]. Norepinephrine indirectly causes epigenetic repression of the ‘protein-kinase-Cε′ gene via ROS production, which increases the heart’s ischemic vulnerability [105]. In the kidneys, norepinephrine indirectly stimulates Na–Cl cotransporter activity by activating basolateral K+ channel activity in the distal convoluted tubule, thereby stimulating Na + absorption [106]. Norepinephrine thus plays a vital role in cognitive function, cardiac ischemic vulnerability, and renal sodium regulation.

NPY, partially released from gut [107], indirectly supports recovery after psychotrauma by reducing social fear, possibly via increased corticosterone (CORT) levels [108]. It indirectly attenuates the consequences of acute myocardial infarction by inhibiting p38/NF-κB-mediated inflammation and fibrosis, reducing the M1 pro-inflammatory macrophages and improving the reparative M2 phenotype, promoting angiogenesis and inhibiting apoptosis [109]. NPY is also indirectly protective against acute kidney injury (AKI) by inhibiting NF-κB-Mincle-mediated M1-macrophage activation and necroinflammation through NPY receptor signalling [110]. Whereas NPY impacts the brain positively via corticosterone, its impact on the heart and kidneys is macrophage-dependent.

Serotonin, which is also produced by the gut microbiota during tryptophan metabolism [111], impacts the brain by controlling motoneuron activity. A moderate release of serotonin (5-HT) onto motoneurons directly boosts motor activity by engaging 5-HT2 receptors, while high levels of 5-HT release lead to overflow to extrasynaptic 5-HT1A receptors on the initial segment of axons, resulting in a decrease of motoneuron activity and an increase of central fatigue [112]. In the heart, serotonin receptor activation directly drives mitosis in valve subendocardial cells [113]. In the kidneys, serotonin receptor variants (5-HT1D, 5-HT1B, and 5-HT7) indirectly induce vasodilation through interaction with cyclooxygenase-derived prostacyclin and ATP-sensitive K^+^ channels [114]. Therefore, serotonin influences motor control in the brain, promotes valvular cell proliferation in the heart, and induces renal vasodilation.

Hormonal gut-liver mediators such as GLP-1, norepinephrine, NPY, and serotonin thus play key roles in regulating metabolism and inflammation while exerting diverse, organ-specific effects on the brain, heart, and kidneys through shared signalling pathways.

### 3.4 Lipids

**Figure 6:**
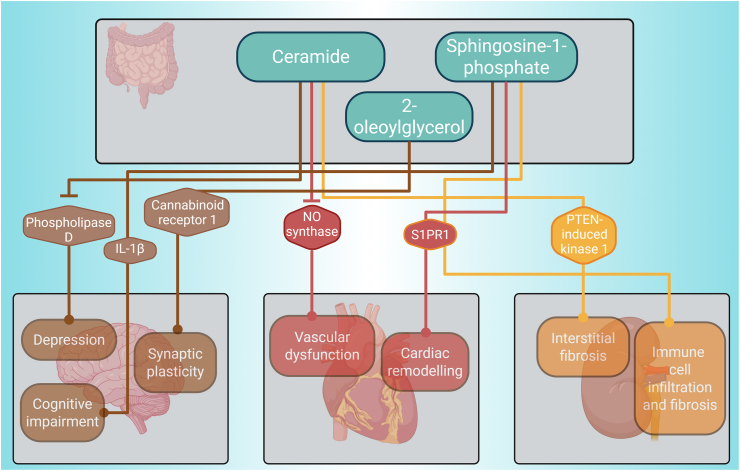
Hormones exert an impact over the brain, heart and kidneys.

The fourth category of gut-liver mediators includes lipids such as 2-oleoylglycerol, ceramide and sphingosine-1-phosphate (S1P), which regulate processes like gluconeogenesis and alcohol-induced liver disease by modulating immune responses and inflammatory pathways [115,116] and their impact on the heart, the brain and the kidneys is depicted in Figure 6. Independent signalling pathways such as intestinal FXR signalling promote ceramide synthesis [115]. Ceramide indirectly contributes to depression by inhibiting phospholipase D activity, decreasing phosphatidic acid levels in the hippocampus [117]. It also indirectly mediates vascular dysfunction by disrupting the endothelial nitric oxide synthase/Akt/heat shock protein 90 signalling complex in the heart [118]. In the kidney, it indirectly exacerbates interstitial fibrosis by interacting with PTEN-induced kinase 1 to inhibit mitophagy of tubular epithelial cells [119]. The ceramide metabolite ‘sphingosine-1-phosphate’ (S1P) is synthesised from ceramide via sphingosine kinases [116]. S1P indirectly causes cognitive impairment through mitochondrial dysfunction and increased IL-1β formation [120]. S1P directly leads to S1PR1 activation in cardiomyocytes, causing a proinflammatory response and exacerbating cardiac remodelling and dysfunction [121]. In kidney perivascular cells, the same mechanism directly stimulates the release of proinflammatory cytokines and chemokines, resulting in immune cell infiltration and fibrosis [122]. Therefore, Ceramide and S1P adversely affect the brain, the heart, and the kidneys. The gut-microbiota produces the lipid 2-oleoylglycerol and mediates 2-oleoylglycerol-induced macrophage priming and subsequent hepatic stellate cell activation [123]. We found less information on the effect of 2-oleoylglycerol. In the brain, it reportedly indirectly mediates synaptic plasticity by antagonising cannabinoid receptor 1 internalization [124]. The impact of 2-oleoylgycerol needs to be studied in more detail before it can be defined as a positive or negative mediator.

### 3.5 Proteins

**Figure 7:**
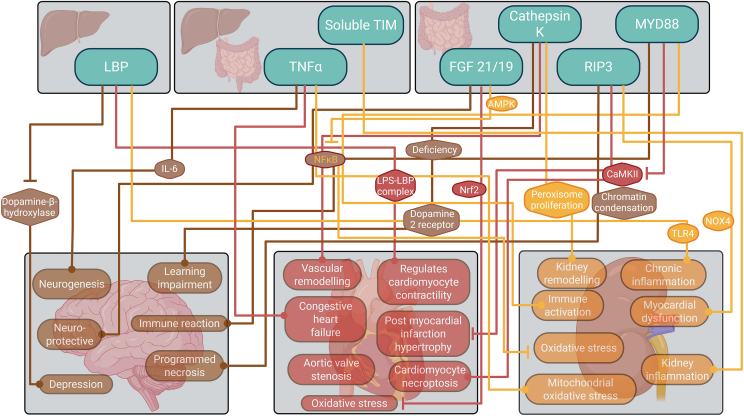
Proteins exert an impact over the brain, heart and kidneys.

The protein mediators retrieved by this search impact liver health through various processes such as inflammation, metabolism and immune response. This group includes (i) Cathepsin K [125], (ii) Fibroblast Growth Factor (FGF) [[126], [127], [128], [129]], (iii) LPS-binding protein (LBP) [130], (iv) myeloid differentiation primary response 88 (MYD88) [131], (v) receptor-interacting protein kinase 3 (RIP3) [132], (vi) soluble TIM3 [133] and (vii) TNF-α [134] and their impact on the heart, the brain and the kidneys is depicted in Figure 7.

Cathepsin K, secreted by colorectal cancer cells [125], has numerous mechanistic roles in the brain, ranging from developing and maintaining the central nervous system to regulating memory and anxiety levels. Cathepsin K deficiency indirectly enhances dopamine levels by upregulating tyrosine hydroxylase, the key enzyme in dopamine biosynthesis. Cathepsin K deficiency also increases dopamine 2-receptor levels, causing learning impairments and reduced anxiety [135]. In the cardiovascular system, disturbed flow leads to an increase in the cathepsin K expression, indirectly contributing to endothelial inflammation and vascular remodelling via integrin αvβ3-cytoskeleton–NF–κB signalling [136]. Blocking the integrin αvβ3-cytoskeleton pathway could effectively inhibit the activation of NF-κB and the expression of cathepsin K [136]. Overexpression of Cathepsin K in kidney mesangial cells indirectly increases peroxisome proliferator-activated receptor-gamma-caspase-8-mediated cell apoptosis, kidney remodelling and hypertension [137]. While Cathepsin K is involved in brain homeostasis, it adversely affects the heart and kidneys.

FGF-15, partially released from ileum [138], indirectly regulates dorsal midbrain neurogenesis and development by controlling the postmitotic transition of dorsal neural progenitors [139]. At the same time, FGF-21 plays a neuroprotective role by regulating the NF-κB and the AMPKα/AKT pathways, decreasing neuroinflammation and oxidative stress [140]. FGF19 indirectly alleviates oxidative stress-induced damage in diabetic hearts by triggering an antioxidant response via AMPK/Nrf2/HO-1 [141]. In addition, FGF-21 also indirectly reduces oxidative stress in the kidneys through AMPK activation, which also inhibits NF-κB-regulated inflammation [142]. Thus, FGF proteins facilitate brain development and have anti-inflammatory effects in the heart and kidneys.

LBP acts indirectly in the brain by promoting depression symptoms by inhibiting monoamine biosynthesis by endogenous dopamine-β-hydroxylase and aromatic-l-amino-acid-decarboxylase [143]. In the heart, LBP directly normalises cardiomyocyte contractility, potentially negating the effects of endotoxin exposure via cluster or micelle-formation of LPS-LBP complexes [144]. LBP indirectly enhances the activation of ‘Toll Like Receptor 4′ signalling, which is crucial in CKD-associated inflammatory response and leads to endothelial dysfunction and chronic inflammation [145]. LBP is expressed in the liver and upregulated through the gut microbiota via ‘myeloid differentiation primary response 88 (MYD88) [131]. Therefore, the gut-liver mediator LBP mediates depression in the brain while rendering other positive effects in the heart and the kidneys.

MYD88 expression indirectly initiates the NF-κB and MAPK pathways, leading to the transcription of inflammatory cytokines and subsequently activating immune cells in both the brain and kidneys [146,147]. MyD88-deficient cardiomyocytes fail to activate NF-κB in the presence of LPS. In addition, the absence of MyD88 indirectly prevents post-myocardial infarction cardiac hypertrophy, inflammation, and oxidised Ca^2+^-calmodulin-dependent protein kinase (CaMKII) expression [148].

RIP3 is constitutively expressed in the intestines [149]. The complex of RIP3 and apoptosis-inducing factors relocates to the nucleus and directly induces chromatin condensation and DNA degradation, initiating programmed necrosis in neurons [150]. In the heart, RIPK3 indirectly induces CaMKII activation by phosphorylation and oxidation, leading to cardiomyocyte necroptosis [151]. RIPK3 also indirectly enhances mitochondrial dysfunction in the kidneys by elevating NOX4 and reducing the expression of subunits of mitochondrial complex I and III [152].

Soluble TIM3 is upregulated in response to severe traumatic brain injury; however, its exact role is currently unknown [153]. It is synthesised by immune cells in both the gut and the liver [154,155]. In coronary heart disease, there is a higher expression of TIM3 in CD4+ T lymphocytes in peripheral blood, along with an increase in the expression of the hematopoietic growth factor IL-7 in blood. However, as in the brain, the exact role of TIM3 or soluble TIM3 in the heart is unknown [156]. In the kidneys, soluble TIM-3 indirectly enhances mitochondrial oxidative stress in cisplatin-induced AKI. Soluble Tim-3 competitively binds to the Tim-3 ligand, accompanied by higher expression of TNF-α, IL-1β and lower expression of IL-10 [157].

Lastly, TNF-α, partially synthesised by both gut and liver [158], negatively regulates embryonic and adult neurogenesis, and promotes myocardial remodelling and kidney inflammation [[159], [160], [161]]. TNF-α indirectly induces the secretion of cytokines of the IL-6 family, such as ‘Leukaemia inhibitory factor’, which plays a crucial role in TNF-α-induced ‘signal transducer and activator of transcription 3’ activation and the development of astrocytes in the brain [159]. TNF-α overexpression directly leads to congestive heart failure [162] while mediating the progression of CKD through apo-A4 expression via the TNF receptor and the 2-NF-κB pathway [161]. Interestingly, TNF-α indirectly protects from worsening cardiac inflammation via induction of cell death of heart-reactive effector CD4+ T cells [163]. Therefore, TNF-α has numerous adverse effects on the brain, the heart and the kidneys.

## 4 Discussion

This review highlights the gut-liver axis as a critical communication pathway through which changes in the intestinal environment influence the brain, the heart and the kidney. A comprehensive study of gut-liver mediators is essential, considering their diverse mechanisms of action in metabolic regulation by impacting the exchange of nutrients, microbial metabolites, and signalling molecules. Disruptions in intestinal microbial balance and barrier function can drive liver inflammation by promoting the translocation of bacterial products. Conversely, hepatic dysfunction can feed back to alter gut physiology, underscoring the bidirectional nature of this relationship. These processes influence energy consumption, inflammatory responses, and the adaptation to environmental factors such as dietary or lifestyle changes or exposure to toxins [164].

This review clearly highlights the gut-liver axis as a central communication network whose perturbations can have far-reaching impacts beyond the liver itself. Disruptions in gut microbial balance and epithelial barrier integrity not only drive liver inflammation but can also influence brain function via the gut–brain axis, contributing to neuroinflammation and cognitive changes. Similarly, altered metabolite profiles originating in the gut and altered bile acid signalling may impair glomerular filtration and promote kidney injury. The heart function is also affected, as microbially derived lipopolysaccharides and trimethylamine N-oxide exacerbate vascular inflammation and arteriosclerosis. This interplay highlights the interconnection of the gut, liver, brain, kidney, and heart.

For example, hormones, like GLP-1 [92] and serotonin [93], and microbial products like indole [29] or butyrate [24], are key players in this interplay. Gut-derived metabolites (especially TMAO and bile-acid panels) add independent predictive power for cardiac events, CKD progression, and neurocognitive decline. By classifying gut-liver-derived mediators retrieved into five specific categories (small molecules, endotoxins, hormones, lipids, and proteins), this review lays the framework for understanding their diverse roles in both health and disease (Figure 2) and their potential impact on the development of novel therapeutic approaches.

### 4.1 Intersecting signalling pathways across the gut-liver-kidney-brain-heart axis

Strikingly, more than 90% of gut-liver mediators identified here affect the kidney, heart and brain; yet most studies focus on them in isolated organs. Prioritising these intersections would yield pathway-centric, cross-organ therapies and more predictive biomarkers and endpoints.

The efficiency of therapeutic discovery for systemic disease can be increased by targeting cross-organ signalling nodes such as NF-κB, NO pathways, TNF-α, dopamine signalling, caspase-3, FXR/TGR5 and CaMKII, which were identified in this manuscript. These signalling pathways collectively shape brain, heart and kidney pathophysiology through multiple mediators simultaneously. At the NF-κB node, the mediator histamine activates microglia via H1/H4-MAPK/PI3K/AKT1-NF-κB, while LPS and LBP drive TLR4-MyD88-NF-κB signalling across organs. The mediator MyD88 is required for NF-κB activation in cardiomyocytes, and the mediator indole constrains neuroinflammation by inhibiting AhR–NF–κB/NLRP3. Also, the mediator NPY suppresses p38/NF-κB in the heart and kidney, with CKD-related endothelial dysfunction also involving TLR4/NF-κB. This highlights the diverse impact of NF-κB across biological processes and organs. Therefore, it is of utmost importance to study the interorgan interlinking mechanisms of the identified therapeutic targets.

NO signalling is likewise intersected: the mediator GLP-1 enhances post-ischaemic cardiac recovery by increasing NO and GLUT-1. The mediator vitamin D induces NO synthase and smooth-muscle relaxation, while the mediator histamine elicits NO-dependent vasodilation and permeability. The mediator LPS elevates NO, whereas ceramide disrupts the eNOS-Akt-Hsp90 complex, highlighting the multifaceted regulation of NO signalling across different organs via multiple mediators. At the TNF-α node, histamine and LPS promote TNF-α release and TNF-α inhibits neurogenesis, driving myocardial remodelling and advancing CKD via TNFR–NF–κB. On the other hand, TNF-α can also delete heart-reactive CD4+ T cells, underscoring organ specificity of the same signalling pathway dependant on a particular mediator in specific organs. Dopamine pathways couple central and peripheral signals. The deficiency of the mediator cathepsin-K elevates dopamine synthesis and D2-receptor levels, while LBP inhibits dopamine-β-hydroxylase and aromatic-l-amino-acid-decarboxylase. Apoptotic control via different mediators converges on caspase-3. The mediator retinoic acid lowers caspase-3 and cytochrome-c-mediated neuronal apoptosis, and in addition, the mediator vitamin D downregulates caspase-3 and Bax in hippocampal neurons. The receptors of the mediator bile acid integrate metabolic-inflammatory control. TGR5 regulates appetite, and in the heart, TGR5 signalling helps explain bile-acid cardio-toxicity versus protection: renal FXR lowers TNF-α/NF-κB and fibrosis (limiting AKI-to-CKD), renal TGR5 upregulates aquaporin-2, and FXR contributes to atherogenesis. The identification of these parallel processes across organs highlights the need to revisit therapeutic targets across organs.

Finally, CaMKII is a death/remodelling node. The mediator RIPK3 activates CaMKII (phosphorylation/oxidation) to drive cardiomyocyte necroptosis, whereas MyD88 deficiency prevents oxidised CaMKII and post–MI hypertrophy/inflammation. Although these mediators are widely studied across diseases and biological processes, their roles have rarely been examined through the lens of multi-organ crosstalk. This gap underscores the urgent need to synthesise the existing literature with inter-organ communication as the central framework of this study.

### 4.2 Clinical implications

Almost all diseases impacted by the gut-liver axis are characterised by chronic inflammation and progressive fibrosis. Emerging gut-centred interventions span a diverse range of modalities: next-generation probiotics designed for durable engraftment, direct administration of microbial metabolites as postbiotics, standardised faecal microbiota transplantation, and carbon-based nanoparticles that adsorb luminal toxins [165]. Complementing these therapies are FXR agonists, TGR5 agonists, GLP-1 receptor antagonists and FGF19 analogues [166]. However, these therapies are so far only focused on the gut-liver axis.

**Figure 8:**
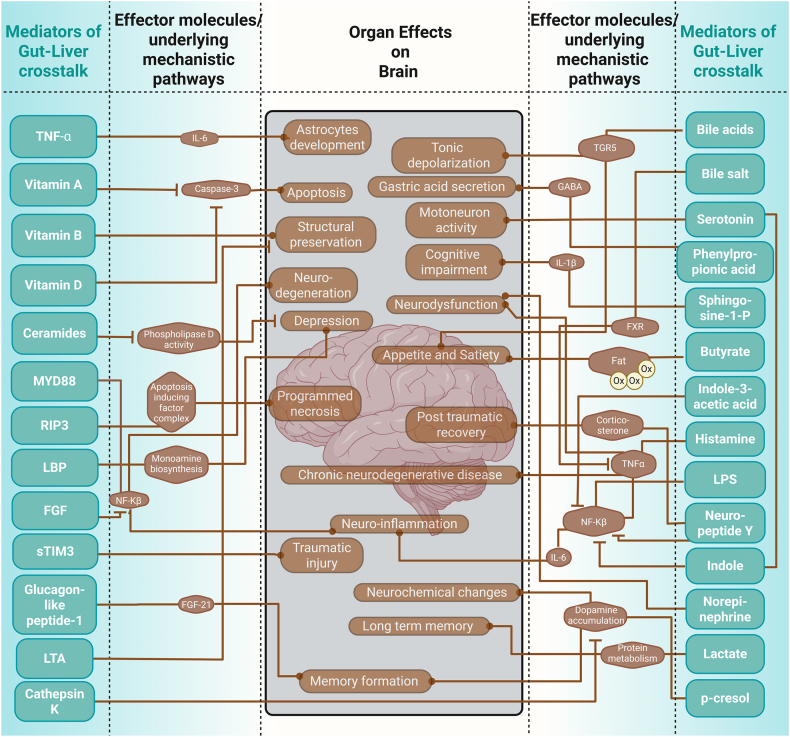
Gut liver mediators exert an influence over the brain.

**Figure 9:**
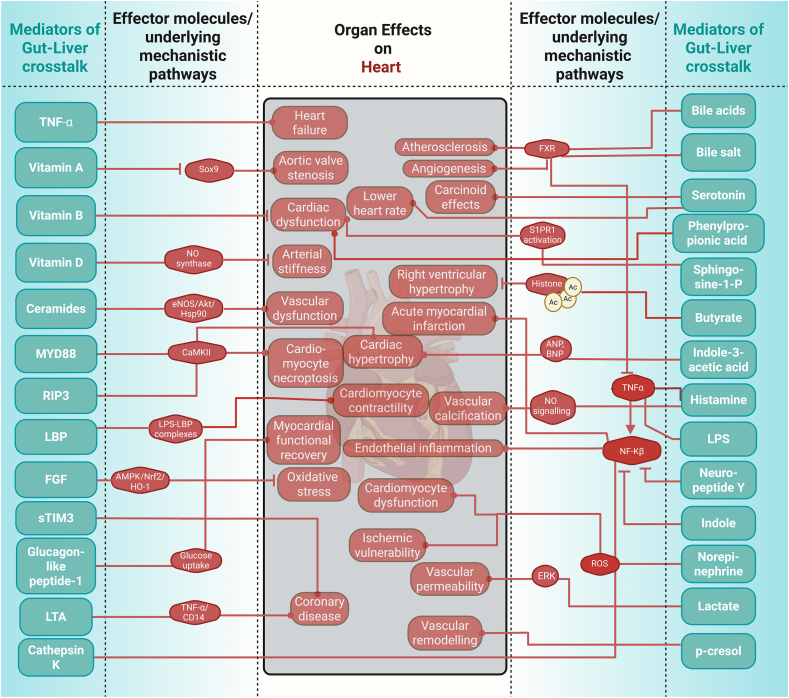
Gut liver mediators exert an influence over the heart.

**Figure 10:**
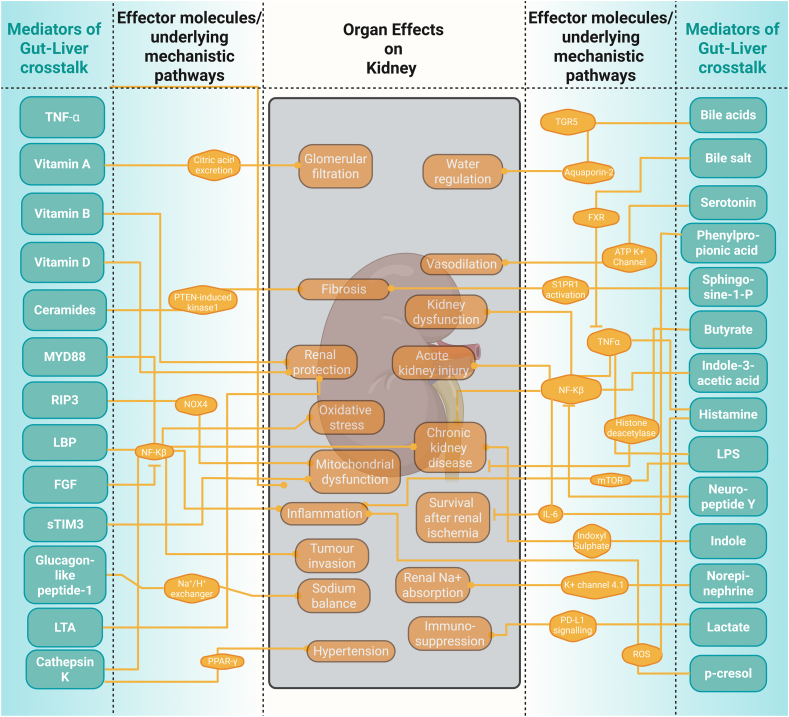
Gut liver mediators exert an influence over the kidneys.

Harmful signals like lipopolysaccharides (LPS) activate the immune pathways that drive tissue injury and remodelling not only in the liver but also in distant organs [167,168]. We investigate how each mediator class influences physiological processes, from metabolic regulation to immune activation, and how their imbalance triggers or exacerbates pathologies of remote organs. In doing so, we identify important mechanistic pathways impacting inter-organ communication with the brain (Figure 8), the heart (Figure 9), and the kidney (Figure 10), as well as novel potential therapeutic strategies, such as modulation of the microbiome, receptor-targeted drugs and enzyme inhibitors that aim to correct specific mediator imbalances. This demonstrates that incorporating multiorgan effects into study designs will enable more precise biomarker discovery and tailored interventions across a range of systemic diseases. Under normal physiological conditions, gut-liver mediators play crucial protective roles by maintaining intestinal barrier integrity, regulating immune tolerance, and fine-tuning metabolic homeostasis. For example, bile acids and short-chain fatty acids support a healthy gut environment, reduce inflammation, and help to control blood sugar and lipid levels [[22], [23], [24], [25], [26]]. These coordinated actions help prevent pathogen translocation, dampen excessive immune responses, and support efficient nutrient processing, thereby safeguarding overall health. Bioactive lipids, including ceramides and sphingosine-1-phosphate, further exacerbate cellular stress and cause damage through pro-inflammatory and apoptotic signalling cascades [115,116]. The convergence of these mediators on common key mechanisms may explain the frequent association of one chronic disease with additional comorbidities over time [169]. Exploring those mediators is key to tackling complex, multi-organ diseases. Modulating bile acid-FXR/TGR5 signalling could improve liver and metabolic function. Boosting butyrate generation supports gut barrier integrity and neuroimmunologic health, and endotoxin neutralisation can prevent TLR4-driven damage across organs. Butyrate might also be a candidate for treating metabolic and neurodegenerative diseases because it enhances energy metabolism and has a neuroprotective effect [170]. Future studies will unravel the specific pathways by which butyrate promotes satiety, enhances energy expenditure, and protects against neuroinflammation.

Therapies modulating histamine pathways, specifically H1R, may help to regulate inflammation and improve outcomes in kidney diseases and neurodegenerative conditions [171,172]; GLP-1 mediates multiple comorbidities, but is particularly interesting as a therapeutic target in vascular diseases induced by diabetes [173]. Further studies on ceramide and sphingosine-1-phosphate as biomarkers for early cognitive decline, cardiac dysfunction, and kidney fibrosis could lead to non-invasive diagnostic tools and uncover therapeutic targets to halt disease progression of multi-organ conditions.

### 4.3 Future perspective

In pathophysiological conditions, the balance between gut-liver mediators is disrupted: mediators, like secondary bile acids or microbial toxins, can provoke inflammation, insulin resistance, or organ damage [174]. This highlights the need for a holistic, systems-level lens rather than isolated treatments impacting individual organs. It is, therefore, imperative that diseases are no longer viewed as isolated conditions but rather as manifestations of systemic dysregulation. By targeting interconnected pathways and mediator networks, interventions can more effectively restore overall physiological balance and prevent downstream complications. Disorders like diabetes, heart disease, and kidney disease often share common underlying mechanisms, like chronic inflammation, oxidative stress, and insulin resistance [175]. These diseases frequently arise together or consecutively and affect each other, largely due to imbalances in inter-organ communication. Integrative therapeutic strategies that address these shared pathways, rather than focusing on a single organ, hold promise for more comprehensive disease prevention and management.

While the review outlines currently known pathways with previously used applied methodologies, a more comprehensive understanding of interorgan communication requires the application of advanced technologies such as multi-omics, systems biology, organ-on-chip platforms, 3D cell cultures, high-resolution imaging, and live multi-organ models. Importantly, many of these technologies are already in development or even available but have not yet or only rarely been applied to study the gut-liver axis and its systemic effects. The integration of multiple tools will enable us to investigate multiple organ systems simultaneously, and will help to uncover complex mediator networks, further deepening our understanding of the mediators consideand allowing us to refine therapeutic strategies.

There is a growing recognition that therapeutic strategies should shift toward multi-target approaches to address a broader network of disease processes. This includes considering the off-label use of existing drugs that act on shared mechanisms such as chronic inflammation, tissue fibrosis, and metabolic dysregulation factors that underlie a wide range of conditions. By aiming to restore systemic balance rather than only treating symptoms or isolated organs, these strategies have the potential to improve long-term outcomes, reduce disease progression, and generate a healthier and happier population.

### 4.4 Limitations

This systematic review is focused on mediators with demonstrated *in vivo* effects in rodent or human studies to ensure biological relevance. However, most of this evidence derives from rodent models that may not fully mirror human physiology, and the diverse experimental methods used across studies can limit direct comparability. Harmonising study designs and incorporating cross-species validation will be crucial in the future for translating these insights into reliable clinical applications. By focusing on five organ systems, we may also have overlooked relevant studies involving other organs like the adipose tissue, pancreas or bone marrow, which also play important roles. This study is focused on the brain, heart, and kidney because of their high shared disease burden and mortality (neurodegeneration, ASCVD/heart failure, CKD). Also, these organs are linked to the gut–liver axis by well-mapped, clinically validated bidirectional pathways. Our analysis examined only unidirectional effects of the gut-liver axis, i.e., its impact on the three other organs, and not the reciprocal effect of heart-kidney-brain conditions on the gut and liver. The search terms were selected based on their relevance to the research objective, which was to capture functional mediators responsible for inter-organ communication, and focusing on evidence derived from experimental (*in vivo*) and clinical studies to ensure translational significance. However, this might have led us to miss the mediators which were not identified as gut-liver mediators in the original manuscripts. Despite these limitations, the data presented in this review provide a clear view of the impact of the gut-liver axis throughout the body and a baseline frame for future multidisciplinary work.

**In summary**, an in-depth investigation of these mediators may indicate their value as early diagnostic and therapeutic targets, not only for intestinal and liver diseases, but also for diseases affecting the brain, heart and kidneys. This broader perspective could lead to more effective, system-wide strategies for managing chronic illness.

## CRediT authorship contribution statement

**Shruti Bhargava:** Writing – review & editing, Writing – original draft, Visualization, Validation, Methodology, Investigation, Formal analysis, Data curation, Conceptualization. **Zhuangting Rao:** Writing – review & editing, Writing – original draft, Methodology, Investigation. **Raymond Vanholder:** Writing – review & editing, Supervision, Formal analysis, Conceptualization. **Frank Tacke:** Writing – review & editing, Supervision, Methodology, Conceptualization. **Heidi Noels:** Writing – review & editing, Validation, Methodology, Funding acquisition, Conceptualization. **Vera Jankowski:** Writing – review & editing, Funding acquisition, Conceptualization. **Juliane Hermann:** Validation. **Joachim Jankowski:** Writing – review & editing, Validation, Supervision, Resources, Project administration, Methodology, Investigation, Funding acquisition, Data curation, Conceptualization.

## Funding

This work was supported by the 10.13039/501100001659German Research Foundation (DFG) Project-ID 322900939 - SFB/TRR219 (to J.J., V.J. and H.N.), Project-ID 403224013 - SFB 1382 (to J.J., F.T. and H.N.) and a grant from the Interdisciplinary Centre for Clinical Research within the Faculty of Medicine at the 10.13039/501100007210RWTH Aachen University (PTD 1-12 to H.N. and V.J.). V.J. is also funded by the 10.13039/100018165CRU 5011 project number 445703531, Cost-Action CA 21165, and ERA-PerMed (ERA-PERMED2022-202-KidneySign).

## Declaration of competing interest

The authors declare the following financial interests/personal relationships which may be considered as potential competing interests: Joachim Jankowski reports was provided by German Research Foundation. Joachim Jankowski reports financial support was provided by European Commission. The authors have no conflict of interest. If there are other authors, they declare that they have no known competing financial interests or personal relationships that could have appeared to influence the work reported in this paper.

## Data Availability

Data will be made available on request.
