# Supplementary material for: The impact of gut-liver-derived mediators on the organ crosstalk with brain, heart, and kidney: A systematic review
Source: Mol Metab. 2025 Nov 29;103:102295. doi: 10.1016/j.molmet.2025.102295 (PMC12765073; doi:10.1016/j.molmet.2025.102295)
Supplement: Multimedia component 1 [file mmc1.docx]

**Supplementary File**

*1^st^ revised version*

**1. Systematic literature search string**

The combination ((gut) AND (liver)) AND (mediator) AND (clinical trial) was used to identify clinical evidence linking gut-liver-derived mediators to systemic outcomes. The combination ((gut) AND (liver)) AND (mediator) AND (in vivo)) was used to include animal or human studies exploring biological mechanisms in a physiological or pathological context. The combination (gut liver axis) AND (clinical trial) was used to identify clinical trials explicitly exploring the gut-liver axis. The combination ((gut-liver axis) AND (mediators) AND (clinical trial)) was used to identify clinical research focusing on signalling molecules within the gut-liver axis. The combination ((gut liver axis) AND (mediators) AND (in vivo)9 was used to identify mechanistic *in vivo* studies investigating the effect of gut-liver mediators on distal organs.

**2. The mechanism by which the identified gut-liver mediators act within the gut-liver axis:**

1. **Low molecular weight intestinal metabolites (MW<1.000Da)**

This category includes low molecular weight intestinal metabolites with a molecular weight below 1,000Da such as (i) bile compounds consisting of bile acids^1-3^, their conjugated products, such as bile salts^4^, and their specific constituents, such as glycocholic-acid^5^, lithocholic acid^6^, and their conjugated isomers^7^, (ii) butyrate^8-12^, (iii) histamine^13^, (iv) indole-3-acetic-acid^14^, (v) indole^15^, (vi) lactate^16-18^, (vii) p-cresol^19^, (viii) phenyl propionic acid (PPA)^20^, (ix) vitamin A^21^, (x) vitamin B 6^22^ and different forms of (xi) vitamin D^23,24^.

The bile compound group classifies mediators derived from cholesterol and synthesised by the liver. Bile compounds are crucial in gut-liver interactions, including intestinal microbial equilibrium, inflammation regulation, cholesterol metabolism modulation, and safeguarding against deleterious metabolic effects^1,2,4-7^. Bile compounds, particularly secondary bile acids, play a role in maintaining a balanced microbial environment in the gut^1^. Diminished concentrations of secondary bile acids in serum and stool are associated with an aberrant microbial balance (dysbiosis), which in turn is correlated with increased serum levels of interleukin-6 (IL-6) ^1^. Moreover, correcting dysbiosis reduces the pro-inflammatory cytokine IL-6 among cirrhosis patients^1^. Additionally, biliary constituents, exemplified by the primary bile acid Muricholic acid, play a pivotal role in cholesterol elimination^2^. Muricholic acid suppresses ´Farnesoid-X-Receptor´ (FXR) signalling in the liver, promoting the efficient elimination of cholesterol and newly synthesised bile acids through faecal and urinary routes, contributing to resistance against hypercholesterolemia, and lowering plasma cholesterol levels by hindering cholesterol absorption by the gut^2^. Bile acids also promote endoplasmic reticulum chaperone 78 kDa glucose‐regulated protein (GRP78) segregation, promoting liver damage^3^.

Moreover, bile salts interact with fibroblast growth factor-19 (FGF-19), leading to alterations in metabolite concentrations and enriching the microbial composition of the gastrointestinal tract^4^. FGF-19 interacts with the bile salt sensor FXR, negatively correlating with pro-inflammatory cytokines like IL-6, protecting against intestinal inflammation^4^. This indicates that bile compounds influence the gut microbiota and inflammation regulation.

However, bile acids also have adverse effects such as insulin resistance, intestinal inflammation and cholestasis^5-7^. Inflammation in the intestine, partially activated through innate activation of monocytes, is a consequence of the bile acid subunit ‘glycocholic-acid’, which significantly correlates with an increase of pro-inflammatory cytokines in the serum, like interferon-gamma (IFN-γ), interleukin-1 β (IL-1β), interleukin-10 (IL-10), interleukin-6 (IL-6), interleukin-12 (IL-12) and tumor necrosis factor-α (TNF-α)^5^. The bile acid ‘lithocholic acid’ is produced by gut microbes and is implicated in the pathogenesis of cholestasis as it promotes repression of bile acid transporters in the liver^6^. The bile acid 'conjugated-isomers-of-linoleic-acids (CLA)', generated by gut microbiota through bacterial isomerase activity, serves as another constituent. CLA functions by downregulating the production peroxisome proliferator-activated receptor gamma (PPAR-γ), promoting fat production and elevating blood sugar levels, ultimately leading to insulin resistance and liver steatosis^7^. Therefore, bile compounds play a dual role in influencing the gut-liver axis, exerting both positive and negative effects, and serving as crucial elements in various physiological interactions between these two organs.

The small molecular weight metabolite, butyrate, mainly produced in the gut^25^, attenuates hepatic steatosis by downregulating genes implicated in lipogenesis and fatty acid degradation^9^. It is involved in lipid and glucose metabolism by enhancing reverse cholesterol transport and stimulating the ‘AMP-activated-protein-kinase’ (AMPK), leading to improvements in insulin sensitivity, oxidative stress response, and a reduction in metabolic dysfunction-associated steatotic liver disease (MASLD) progression. Butyrate also improves the function of the intestinal barrier^8,10^ and induces apoptosis of proinflammatory macrophages specifically^11^. Treatment with butyrate alleviates immune-mediated cholangitis by rescuing the impaired function of myeloid-derived suppressor cells^12^.

The endogenously produced low molecular weight metabolite ‘histamine’, produced in the gut with receptors in the liver^26^, reduces plasma insulin-like-growth-factor-I (IGF-1) via the histamine-H1-receptor-mediated pathway, resulting in tissue repair and wound healing^13^.

Metabolites ‘indole’ and ‘indole-3-acetic-acid’, often produced by gut microbiota^27^, eliminate liver inflammation by reducing the production of pro-inflammatory ‘nuclear factor-kappa-light-chain-enhancer of activated B-cell (NF-κβ)-responsive genes’ and key mediators of the NOD-like receptor family pyrin domain containing 3 (NLRP3) inflammasome pathway^14^. In addition, indole-3-acetic acid reduces the expression of pro-inflammatory cytokines by decreasing the infiltration of activated macrophages into liver tissue^14^. Indole also protects against lipopolysaccharide (LPS)-induced alterations in cholesterol metabolism through transcriptional regulation associated with increased hepatic levels of 4β-hydroxycholesterol^15^.

Due to dysbiosis, the intestinal microbiomes produce excess lactate, which activates nicotinamide-adenine-dinucleotide phosphate-oxidase-2 (NOX2) in the intestine, leading to the production of reactive oxygen species (ROS). These ROS transform the latent form of transforming growth factor- β (TGF-β) into the active form, which subsequently triggers the formation of myofibroblast cell phenotypes, extracellular matrix protein deposition, and fibrosis in the intestine^16,17^. Lactate, particularly the D stereoisomer, promotes the capture, clearance, and killing of pathogens by liver-resident Kupffer cells, but lactate-linked redox activation of NOX2 is also known to induce ectopic inflammatory foci in the liver^16,17^. Finally, D-lactate guides neutrophil homing to the liver^28^, and inhibits autophagy in hepatocytes by inhibiting inflammation caused by NLRP3 inflammasome^18^.

The low molecular weight metabolite ‘p-cresol’ regulates blood glucose levels and enhances pancreatic function while downregulating the expression of pro-inflammatory cytokines such as TNF-α, IL-6, and IL-10. ‘P-cresol’ upregulates sirtuin 1, promoting insulin secretion and lipolysis, reducing adipogenesis, preventing liver lipid accumulation and inflammation, and ultimately improving glucose homeostasis^19^. PPA produced by gut microbes reduces the expression of cytochrome P450 2E1 (CYP2E1) in the liver. CYP2E1 catalyses the conversion of acetaminophen (APAP) to a reactive, toxic metabolite, which leads to hepatic injury^20^.

Vitamins A, B and D reduce inflammation and preserve intestinal structure. By converting vitamin A into retinoic acid, liver sinusoidal endothelial cells prime CD4+ T-cells for migrating from the liver to the gut, potentially reducing intestinal inflammation^21^. The deficiency of vitamin B6, which is partly produced by gut microbiota, leads to ornithine aminotransferase expression inhibition, leading to oxidative stress and dysregulated amino acid metabolism in the liver^22^. 1,25-(OH)2D3, active form of vitamin D, maintains the intestinal mucosal barrier, inhibits TNFα-NF-κβ signals, and normalizes tight-junction protein expression, counteracting chronic mucosal inflammation counteracting chronic mucosal inflammation and upregulating intestinal epithelial vitamin-D receptors, increasing TNF-α inhibition^23^. Liver produces 25-hydroxyvitamin-D^29^ and the metabolite D3-3β-glucuronic acid of vitamin-D-25-Hydroxyvitamin stimulates the vitamin-D-receptor-mediated pathway in the colon, primarily through apical entry after cleavage by gut bacteria, triggering the expression of vitamin-D-dependent genes involved in calcium absorption and colon cell health. This mechanism suggests a significant role for enterohepatic signalling in regulating calcium absorption and maintaining colon epithelial function^24^.

**The low molecular weight metabolites identified via this systematic literature search mostly exert beneficial effects on the gut-liver axis,** **primarily playing a crucial role in preventing inflammation along this axis.**

1. **Endotoxins**

The second category of gut-liver mediators belongs to the group of **endotoxins**, predominantly lipopolysaccharides (LPS) and lipoteichoic acid (LTA). LPS plays a central role in initiating and propagating inflammation in the liver, contributing to liver damage and fibrosis^30,31^. Intestinal LPS use lipid uptake pathways in the small intestine, regulated by neurohormones, to gain access to the liver through the portal vein^32^. Gut-derived LPS also has a direct impact on pancreatic stellate cells, which respond by upregulating the expression of Toll-like receptor 4 (TLR4), triggering an inflammatory response^33,34^. The inflammatory response is induced by stimulating the production of pro-inflammatory cytokines, including IL-1, IL-6, and TNF-α, and IL-10 by monocytes and macrophages, with IL-10 downregulating granulocyte activation, IFN-γ and IL-12 release, and adhesion molecule expression, crucial for modulating inflammation^30,31^. Importantly, endotoxins also trigger the production of ‘nitric oxide’ (NO), modulating inflammation by hindering TNF-α-mediated inflammatory processes^30^. In addition, LTA concentration increases in advanced chronic liver disease and is correlated to renin levels but not to inflammatory markers in these patients^35^.

1. **Hormones**

The third category of retrieved gut-liver mediators includes (i) glucagon-like peptide-1 (GLP-1)^36^, (ii) serotonin^37^, (iii) norepinephrin^38^, (iv) neuropeptide Y (NPY)^39^, (v) metabolitin^40^. The hormone GLP-1, produced by specialised intestinal neuroendocrine cells in response to dietary components^41^, exerts anti-lipogenic and anti-inflammatory effects in the liver by promoting the activation of AMPK through the induction of 'cyclic-adenosine-monophosphate' (cAMP)^36^. This activation leads to a shift in liver metabolism from carbohydrates to fat breakdown. AMPK, in turn, inhibits lipogenic enzymes and downregulates transcription factors related to lipid metabolism. Additionally, GLP-1 promotes the activation of Carnitine palmitoyltransferase I (CPT-1), a key enzyme in fatty acid beta-oxidation^36^. These effects help mitigate hepatic steatosis and promote liver health^36^. Another hormone that operates via cAMP induction is serotonin, which is also produced by the gut microbiota during tryptophan metabolism^42^ and plays a role in liver regeneration. Serotonin activates the ´cAMP‐coupled 5‐hydroxytryptamine-7´ receptor on hepatocytes, triggering protein kinase A/ cAMP response element binding protein phosphorylation/AKT activation and IGF-1 secretion in tumor microenvironments^37^. The third hormone, norepinephrine, also influences the gut-liver axis through cAMP. It is in part produced by the gut microbes^26^. In early sepsis stages, norepinephrine induces liver cell dysfunction by activating α2-adrenergic receptors and stimulating Kupffer cells, thereby promoting the release of TNF-α and the cytokines IL-1β and IL-6^38^. The α2-adrenoreceptor stimulates Kupffer cells by the inhibition of adenylate cyclase via the inhibitory guanosine-5'-triphosphate binding protein subunit and subsequent suppression of intracellular cAMP and consequent increase of TNF-α production, aggravating sepsis-related liver dysfunction^38^.

Neuropeptide Y (NPY), a sympathetic co-transmitter of norepinephrine, reduces portal hypertension in cirrhotic conditions. NPY enhances vascular contractility by increasing the expression and activity of Rho-kinase and reducing NO-mediated vasorelaxation and arterial vasodilation in mesenteric arteries^39^.

Metabolitin (MTL), a recently identified circulating pentadecapeptide, regulates fat and glucose metabolism by interacting with the G-protein coupled receptor 6A in the intestine^40^. MTL inhibits the de novo synthesis of lipids in the liver by acting on the acetyl-CoA carboxylase pathway. Simultaneously, it suppresses neurotensin secretion, inhibiting triglyceride absorption in the gut through the 5′AMP-activated protein kinase pathway^40^. In addition, MTL enhances the expression of GLP-1 in the intestine. This regulation of neurotensin and GLP-1 leads to reduced fat absorption, improved insulin secretion, and blood glucose stabilization, leading to improvement of non-alcoholic fatty liver disease  (NAFLD) and insulin sensitivity^40^.

1. **Lipids**

The fourth category of gut-liver mediators includes lipids such as 2-oleoylglycerol, ceramide and sphingosine-1-phosphate. The gut-microbiota produces the lipid 2-oleoylglycerol and mediates 2-oleoylglycerol-induced macrophage priming and subsequent hepatic stellate cell activation via the ‘G Protein-Coupled Receptor 119/Transforming Growth Factor-β-Activated Kinase 1/NF-κB/transforming growth factor-β1 (TGF-β1)’ signalling pathway, leading to liver inflammation and hepatic fibrosis^43^.

In addition, independent signalling pathways such as intestinal FXR signalling promote ceramide synthesis. Ceramides increase hepatic gluconeogenesis and glucose metabolism by inhibiting the liver mitochondrial citrate synthase and increasing mitochondrial acetyl-CoA levels, inducing endoplasmic reticulum stress and calcium influx^44^. The ceramide metabolite ´sphingosine-1-phosphate´ (S1P) is synthesised from ceramide via sphingosine kinases45, the activity of which, through activation of retinoid-related orphan receptor gamma, contributes to the proliferation of Th17 cells, leading to alcoholic gut inflammation, steatosis and liver damage. Sphingosine kinase-2 deficiency is correlated with NF-κβ activation, IL-6 and TNF-α formation and sphingosine-1-phosphate receptor 1 (S1PR1) expression, emphasising the necessity of further understanding these mediators in the gut-liver axis^45^.

1. **Proteins**

The fifth category of mediators consists of proteins such as (i) Cathepsin K^46^, (ii) Fibroblast Growth Factor (FGF)^47-50^, (iii) LPS-binding protein (LBP)^51^, (iv) myeloid differentiation primary response 88´ (MYD88) ^52^, (v) receptor-interacting protein kinase 3 (RIP3)^53^, (vi) soluble ´mucin domain-containing protein-3´ (TIM3)^54^ and (vii) TNF^55^. Cathepsin K, secreted by colorectal cancer cells, promotes the polarization of tumour-associated macrophages by binding to TLR4 to stimulate the mTOR-dependent pathways^46^. The effect of Cathepsin K is driven by IL-10 and interleukin-17 (IL-17), which, in turn, promote the invasion and metastasis of colorectal cancer cells through the NF-κβ pathway^46^. FGFs maintain lipid metabolism and intestinal structures. Fibroblast growth factors-15/19 (FGF-15/19) modulate liver fat metabolism and protect the liver from lipid-mediated cellular stress and injury by mediating adipogenesis associated with downregulation of transcription factors like ´peroxisome proliferator-activated receptor gamma 2´, involved in upregulating adipogenic genes such as CD36 and Mogat1. FGF-15/19 facilitates liver regeneration through mitosis, cell protection, and stimulation of protein synthesis^47^. FGF-15/19 also interact with other mediators, such as bile acids, and their deficiency leads to an increase in bile acid levels, which subsequently enhances FXR signalling in hematopoietic stem cells, resulting in reduced liver fibrosis^48,50^. ´Fibroblast growth factor-21´ (FGF-21), a member of the FGF-19 subfamily, significantly lowers steatohepatitis and collagen deposition while restoring intestinal structure. Additionally, FGF-21 alters gut microbiota composition and influences bile acid metabolism, ultimately regulating dysbiosis and alleviating MASLD^49^. ´LPS-binding protein´ (LBP) hampers insulin signalling within hepatocytes and systemic glucose metabolism^51^. LBP is expressed in the liver and upregulated through the gut microbiota via ´myeloid differentiation primary response 88´ (MYD88), an inflammatory gene^52^, exemplifying the role of gut-liver mediators produced by microbes^51^. While LBP interacts with LPS, the protein ‘receptor-interacting protein kinase 3´ (RIP3) is activated by LPS, leading to the RIP3 signalling pathway, causing plasma membrane permeabilisation with pro-inflammatory effects^53^. RIP3 inhibition dampens the activation and accumulation of liver macrophages, thus enhancing liver injury^53^.

Membrane-bound ‘T cell immunoglobulin and mucin-domain containing-3’ (TIM3), of which the efficacy is modulated by gut microbiota^56^, leads to functional inactivation of immune cells when engaged by their ligands ´Galectin-9´ and ´carcinoembryonic antigen-related cell adhesion molecule 1´ (CEACAM1). Soluble TIM3 acts as a decoy antagonist and sequesters Galectin-9 and CEACAM1, potentially preventing immune suppression in alcoholic liver disease^54^.

Lastly, tumour necrosis factor (TNF) enhances glucose uptake in the gut, liver and elsewhere and induces insulin resistance by reducing the number or activity of the insulin-sensitive ´glucose transporter type-4’ (GLUT-4) isoforms and by down-regulating the translocation of GLUT-4 from its intracellular location to the cell membrane^55^.

**References:**

1. Bajaj JS, Salzman N, Acharya C, et al. Microbial functional change is linked with clinical outcomes after capsular fecal transplant in cirrhosis. *JCI insight*. 2019;4(24)

2. Gaillard D, Masson D, Garo E, et al. Muricholic acids promote resistance to hypercholesterolemia in cholesterol-fed mice. *International Journal of Molecular Sciences*. 2021;22(13):7163.

3. Spivak I, Guldiken N, Usachov V, et al. Alpha-1 Antitrypsin Inclusions Sequester GRP78 in a Bile Acid-Inducible Manner. *Liver Int*. Jan 2025;45(1):e16207. doi:10.1111/liv.16207

4. Koelfat KV, Picot D, Chang X, et al. Chyme Reinfusion Restores the Regulatory Bile Salt–FGF19 Axis in Patients With Intestinal Failure. *Hepatology*. 2021;74(5):2670-2683.

5. Zhao X, Setchell KD, Huang R, et al. Bile acid profiling reveals distinct signatures in undernourished children with environmental enteric dysfunction. *The Journal of Nutrition*. 2021;151(12):3689-3700.

6. Owen BM, Milona A, van Mil S, et al. Intestinal detoxification limits the activation of hepatic pregnane X receptor by lithocholic acid. *Drug metabolism and disposition*. 2010;38(1):143-149.

7. Druart C, Neyrinck AM, Dewulf EM, et al. Implication of fermentable carbohydrates targeting the gut microbiota on conjugated linoleic acid production in high-fat-fed mice. *British journal of nutrition*. 2013;110(6):998-1011.

8. Endo H, Niioka M, Kobayashi N, Tanaka M, Watanabe T. Butyrate-producing probiotics reduce nonalcoholic fatty liver disease progression in rats: new insight into the probiotics for the gut-liver axis. *PloS one*. 2013;8(5):e63388.

9. Du Y, Li X, Su C, et al. Butyrate protects against high‐fat diet‐induced atherosclerosis via up‐regulating ABCA1 expression in apolipoprotein E‐deficiency mice. *British Journal of Pharmacology*. 2020;177(8):1754-1772.

10. Zhao S, Zhang H, Zhu H, et al. Gut microbiota promotes macrophage M1 polarization in hepatic sinusoidal obstruction syndrome via regulating intestinal barrier function mediated by butyrate. *Gut Microbes*. Jan-Dec 2024;16(1):2377567. doi:10.1080/19490976.2024.2377567

11. Sarkar A, Mitra P, Lahiri A, et al. Butyrate limits inflammatory macrophage niche in NASH. *Cell Death Dis*. May 18 2023;14(5):332. doi:10.1038/s41419-023-05853-6

12. Wang R, Li B, Huang B, et al. Gut Microbiota-Derived Butyrate Induces Epigenetic and Metabolic Reprogramming in Myeloid-Derived Suppressor Cells to Alleviate Primary Biliary Cholangitis. *Gastroenterology*. Sep 2024;167(4):733-749 e3. doi:10.1053/j.gastro.2024.05.014

13. Liao W, Rudling M, Möller C, Angelin B. Endogenous histamine reduces plasma insulin-like growth factor I via H1 receptor-mediated pathway in the rat. *European journal of pharmacology*. 1999;374(3):471-476.

14. Ji Y, Gao Y, Chen H, Yin Y, Zhang W. Indole-3-acetic acid alleviates nonalcoholic fatty liver disease in mice via attenuation of hepatic lipogenesis, and oxidative and inflammatory stress. *Nutrients*. 2019;11(9):2062.

15. Beaumont M, Neyrinck AM, Olivares M, et al. The gut microbiota metabolite indole alleviates liver inflammation in mice. *The FASEB Journal*. 2018;32(12):6681.

16. Sarkar S, Saha P, Seth RK, et al. Higher intestinal and circulatory lactate associated NOX2 activation leads to an ectopic fibrotic pathology following microcystin co-exposure in murine fatty liver disease. *Comparative Biochemistry and Physiology Part C: Toxicology & Pharmacology*. 2020;238:108854.

17. McDonald B, Zucoloto AZ, Yu I-L, et al. Programing of an intravascular immune firewall by the gut microbiota protects against pathogen dissemination during infection. *Cell host & microbe*. 2020;28(5):660-668. e4.

18. Zhao H, Zhou J, Yuan L, et al. Exploring the alleviating effects of Bifidobacterium metabolite lactic acid on non-alcoholic steatohepatitis through the gut-liver axis. *Front Microbiol*. 2024;15:1518150. doi:10.3389/fmicb.2024.1518150

19. Brial F, Alzaid F, Sonomura K, et al. The natural metabolite 4-cresol improves glucose homeostasis and enhances β-cell function. *Cell reports*. 2020;30(7):2306-2320. e5.

20. Cho S, Yang X, Won KJ, et al. Phenylpropionic acid produced by gut microbiota alleviates acetaminophen-induced hepatotoxicity. *Gut Microbes*. Jan-Dec 2023;15(1):2231590. doi:10.1080/19490976.2023.2231590

21. Neumann K, Kruse N, Szilagyi B, et al. Connecting liver and gut: murine liver sinusoidal endothelium induces gut tropism of CD4+ T cells via retinoic acid. *Hepatology*. 2012;55(6):1976-1984.

22. Shen H, Zhou L, Yang Y, et al. The gut microbiota-produced vitamin B6 mitigates alcohol-associated liver disease by attenuating hepatic oxidative stress damage. *Hepatol Commun*. Jan 1 2025;9(1)doi:10.1097/HC9.0000000000000599

23. Su Y-B, Li T-H, Huang C-C, et al. Chronic calcitriol supplementation improves the inflammatory profiles of circulating monocytes and the associated intestinal/adipose tissue alteration in a diet-induced steatohepatitis rat model. *PloS one*. 2018;13(4):e0194867.

24. Reynolds CJ, Koszewski NJ, Horst RL, Beitz DC, Goff JP. Role of glucuronidated 25-hydroxyvitamin D on colon gene expression in mice. *American Journal of Physiology-Gastrointestinal and Liver Physiology*. 2020;319(2):G253-G260.

25. Beyer-Sehlmeyer G, Glei M, Hartmann E, et al. Butyrate is only one of several growth inhibitors produced during gut flora-mediated fermentation of dietary fibre sources. *Br J Nutr*. Dec 2003;90(6):1057-70. doi:10.1079/bjn20031003

26. Dicks LMT. Gut Bacteria and Neurotransmitters. *Microorganisms*. Sep 14 2022;10(9)doi:10.3390/microorganisms10091838

27. Sun J, Zhang Y, Kong Y, et al. Microbiota-derived metabolite Indoles induced aryl hydrocarbon receptor activation and inhibited neuroinflammation in APP/PS1 mice. *Brain Behav Immun*. Nov 2022;106:76-88. doi:10.1016/j.bbi.2022.08.003

28. Zucoloto AZ, Schlechte J, Ignacio A, et al. Vascular traffic control of neutrophil recruitment to the liver by microbiota-endothelium crosstalk. *Cell Rep*. May 30 2023;42(5):112507. doi:10.1016/j.celrep.2023.112507

29. Borel P, Caillaud D, Cano NJ. Vitamin D bioavailability: state of the art. *Crit Rev Food Sci Nutr*. 2015;55(9):1193-205. doi:10.1080/10408398.2012.688897

30. McCuskey R, Nishida J, Eguchi H, et al. Role of endotoxin in the hepatic microvascular inflammatory response to ethanol. *Journal of gastroenterology and hepatology*. 1995;10(S1):S18-S23.

31. Von Baehr V, Döcke W, Plauth M, et al. Mechanisms of endotoxin tolerance in patients with alcoholic liver cirrhosis: role of interleukin 10, interleukin 1 receptor antagonist, and soluble tumour necrosis factor receptors as well as effector cell desensitisation. *Gut*. 2000;47(2):281-287.

32. Akiba Y, Maruta K, Takajo T, et al. Lipopolysaccharides transport during fat absorption in rodent small intestine. *American Journal of Physiology-Gastrointestinal and Liver Physiology*. 2020;318(6):G1070-G1087.

33. Vonlaufen A, Xu Z, Daniel B, et al. Bacterial endotoxin: a trigger factor for alcoholic pancreatitis? Evidence from a novel, physiologically relevant animal model. *Gastroenterology*. 2007;133(4):1293-1303.

34. Lin Y, Yu L-X, Yan H-X, et al. Gut-Derived Lipopolysaccharide Promotes T-Cell–Mediated Hepatitis in Mice through Toll-Like Receptor 4Gut-Derived LPS Promote Con A–Induced Hepatitis. *Cancer Prevention Research*. 2012;5(9):1090-1102.

35. Simbrunner B, Caparros E, Neuwirth T, et al. Bacterial translocation occurs early in cirrhosis and triggers a selective inflammatory response. *Hepatol Int*. Aug 2023;17(4):1045-1056. doi:10.1007/s12072-023-10496-y

36. Ben-Shlomo S, Zvibel I, Shnell M, et al. Glucagon-like peptide-1 reduces hepatic lipogenesis via activation of AMP-activated protein kinase. *Journal of hepatology*. 2011;54(6):1214-1223.

37. Svejda B, Kidd M, Timberlake A, et al. Serotonin and the 5‐HT 7 receptor: The link between hepatocytes, IGF‐1 and small intestinal neuroendocrine tumors. *Cancer science*. 2013;104(7):844-855.

38. Yang S, Zhou M, Chaudry IH, Wang P. Norepinephrine-induced hepatocellular dysfunction in early sepsis is mediated by activation of α2-adrenoceptors. *American Journal of Physiology-Gastrointestinal and Liver Physiology*. 2001;

39. Moleda L, Trebicka J, Dietrich P, et al. Amelioration of portal hypertension and the hyperdynamic circulatory syndrome in cirrhotic rats by neuropeptide Y via pronounced splanchnic vasoaction. *Gut*. 2011;60(8):1122-1132.

40. Teng B, Huang C, Cheng C-L, et al. Newly identified peptide hormone inhibits intestinal fat absorption and improves NAFLD through its receptor GPRC6A. *Journal of hepatology*. 2020;73(2):383-393.

41. Puddu A, Sanguineti R, Montecucco F, Viviani GL. Glucagon-like peptide-1 secreting cell function as well as production of inflammatory reactive oxygen species is differently regulated by glycated serum and high levels of glucose. *Mediators Inflamm*. 2014;2014:923120. doi:10.1155/2014/923120

42. Agus A, Planchais J, Sokol H. Gut Microbiota Regulation of Tryptophan Metabolism in Health and Disease. *Cell Host Microbe*. Jun 13 2018;23(6):716-724. doi:10.1016/j.chom.2018.05.003

43. Yang M, Qi X, Li N, et al. Western diet contributes to the pathogenesis of non-alcoholic steatohepatitis in male mice via remodeling gut microbiota and increasing production of 2-oleoylglycerol. *Nat Commun*. Jan 16 2023;14(1):228. doi:10.1038/s41467-023-35861-1

44. Xie C, Jiang C, Shi J, et al. An intestinal farnesoid X receptor–ceramide signaling axis modulates hepatic gluconeogenesis in mice. *Diabetes*. 2017;66(3):613-626.

45. Chu S, Sun R, Gu X, et al. Inhibition of sphingosine‐1‐phosphate‐induced Th17 cells ameliorates alcohol‐associated steatohepatitis in mice. *Hepatology*. 2021;73(3):952-967.

46. Li R, Zhou R, Wang H, et al. Gut microbiota-stimulated cathepsin K secretion mediates TLR4-dependent M2 macrophage polarization and promotes tumor metastasis in colorectal cancer. *Cell Death & Differentiation*. 2019;26(11):2447-2463.

47. Alvarez-Sola G, Uriarte I, Latasa MU, et al. Fibroblast growth factor 15/19 (FGF15/19) protects from diet-induced hepatic steatosis: development of an FGF19-based chimeric molecule to promote fatty liver regeneration. *Gut*. 2017;66(10):1818-1828.

48. Schumacher JD, Kong B, Wu J, et al. Direct and indirect effects of fibroblast growth factor (FGF) 15 and FGF19 on liver fibrosis development. *Hepatology*. 2020;71(2):670-685.

49. Lin D, Sun Q, Liu Z, et al. Gut microbiota and bile acids partially mediate the improvement of fibroblast growth factor 21 on methionine-choline-deficient diet-induced non-alcoholic fatty liver disease mice. *Free Radical Biology and Medicine*. 2023;195:199-218.

50. Simbrunner B, Hofer BS, Schwabl P, et al. FXR-FGF19 signaling in the gut-liver axis is dysregulated in patients with cirrhosis and correlates with impaired intestinal defence. *Hepatol Int*. Jun 2024;18(3):929-942. doi:10.1007/s12072-023-10636-4

51. Molinaro A, Koh A, Wu H, et al. Hepatic expression of lipopolysaccharide-binding protein (Lbp) is induced by the gut microbiota through Myd88 and impairs glucose tolerance in mice independent of obesity. *Molecular Metabolism*. 2020;37:100997.

52. Wang Y, Lv B, Liu N, et al. The mechanism of bile acid metabolism regulating lipid metabolism and inflammatory response in T2DM through the gut-liver axis. *Heliyon*. Aug 30 2024;10(16):e35421. doi:10.1016/j.heliyon.2024.e35421

53. Zhang H, Liu M, Zhong W, et al. Leaky gut driven by dysbiosis augments activation and accumulation of liver macrophages via RIP3 signaling pathway in autoimmune hepatitis. *Frontiers in Immunology*. 2021;12:624360.

54. Riva A, Palma E, Devshi D, et al. Soluble TIM3 and its ligands galectin-9 and CEACAM1 are in disequilibrium during alcohol-related liver disease and promote impairment of anti-bacterial immunity. *Frontiers in Physiology*. 2021;12:632502.

55. Lang CH, Dobrescu C, Bagby GJ. Tumor necrosis factor impairs insulin action on peripheral glucose disposal and hepatic glucose output. *Endocrinology*. 1992;130(1):43-52.
